# Phytoplankton cell size control can be affected by photosynthetic light energy utilization

**DOI:** 10.3389/fmicb.2022.1008606

**Published:** 2022-11-02

**Authors:** Wanzhu Li, Baoli Wang, Jing Xiao, Meiling Yang, Sheng Xu, Cong-Qiang Liu

**Affiliations:** Institute of Surface-Earth System Science, School of Earth System Science, Tianjin University, Tianjin, China

**Keywords:** cell size, light energy conversion, metatranscriptome, molecular regulatory mechanism, phytoplankton, transcriptome

## Abstract

Phytoplankton cell size is well known as an essential functional trait, but its control factors are still unclear. Considering light provides the necessary energy for phytoplankton survival, we hypothesized that photosynthetic light energy utilization could influence phytoplankton cell size control. Several scenarios were conducted to understand the relationship between *F_v_*/*F_m_* and cell size for phytoplankton interspecies, and metatranscriptome in the field and transcriptome in the laboratory were used to understand relevant molecular mechanisms. The results indicated that there was a universal significant positive relationship between *F_v_*/*F_m_* and cell volume in general. The molecular evidence demonstrated that light utilization by phytoplankton regulates their cell size by harmonizing the generation and allocation of chemical energy and fixed carbon in the cell. Phytoplankton cell size would cease to enlarge once the increased light energy conversion and subsequent fixed carbon could no longer satisfy the increasing demand of size enlargement. This unity of energy and matter in shaping phytoplankton size results in cell size being an important functional trait. This study is the first to discover the above molecular mechanisms and is helpful to deepen the understanding on the cell size control of phytoplankton.

## Introduction

Phytoplankton are primary producers and play an important role in aquatic ecosystems (Karlusich et al., 2020). Phytoplankton cell size, varying widely from less than 1 to 10^9^ μm^3^ (Beardall et al., 2009), is well known as an essential functional trait determining the performance at species and community levels (Maranon, 2015; Hillebrand et al., 2022a). As such, phytoplankton cell size has an allometric relationship with the functional performance, including cellular rates of carbon fixation, respiration, and exudation as well as resource uptake and content (Maranon et al., 2013; Hillebrand et al., 2022b). On the other hand, phytoplankton cell size can be influenced by grazing and environmental stress such as nutrient limitation and warming (Kiørboe, 1993; Maranon et al., 2013). However, when referring to the problem of what determines cell size of phytoplankton, it remains unclear.

Photosynthesis, an essential performance of phytoplankton, converts light energy into chemical energy, later driving cellular metabolism (Blankenship, 2014). Light provides the necessary energy for phytoplankton survival and could constrain their cell size. Current studies in molecular biology demonstrate that cytoskeleton plays an important role in cell size control (Fletcher and Mullins, 2010; Marshall et al., 2012). Carbon is a major component of the above organelle (Ochs et al., 2014; Finkel et al., 2016) and as material source, should involve in the size control. Therefore, we hypothesized that photosynthetic light energy utilization could affect phytoplankton cell size control by regulating cellular carbon demand.

The capture of light energy in a phytoplankton cell occurs mainly in Photosystem II (PS II) (Fischer et al., 2016). The maximum quantum yield of PS II (i.e., *F_v_*/*F_m_*) is the maximal efficiency by which PS II turns energy in absorbed photons into electron flow and is widely used to indicate photosynthetic light energy utilization (Malerba et al., 2018; Yan et al., 2018; Tan et al., 2019). Several scenarios were designed to verify the proposed hypothesis. In the laboratory, to understand the relationship between *F_v_*/*F_m_* and cell size among phytoplankton interspecies, twenty-three algal species were cultured under normal condition, and the published data of *F_v_*/*F_m_* in other experimental phytoplankton (including freshwater and seawater species) were collected and integrated. In addition, *Chlamydomonas reinhardtii* FACHB-479 (*C. reinhardtii*) was used as a representative to understand how photosynthetic light energy utilization regulates the cell size by analyzing its transcriptome, and significant difference in *F_v_*/*F_m_* of *C. reinhardtii* was artificially created by nutrient limitation. In the field, seven reservoirs in Tianjin, North China, were investigated to understand the *F_v_*/*F_m_* and mean cell size of phytoplankton assemblage and related environmental factors, and metatranscriptome analyses were conducted to understand the relevant molecular regulation at community level. In addition, seasonal survey of three reservoirs on the Wujiang River, Southwest China, were conducted to understand the relationship between *F_v_*/*F_m_* and mean cell size for phytoplankton assemblage. The main aim of this study was to understand whether there is a universal relationship between *F_v_*/*F_m_* and cell size for phytoplankton and the molecular regulation mechanisms that light energy utilization regulates cell size. This study is helpful to deepen the understanding on the cell size control of phytoplankton.

## Materials and methods

### Laboratory culture experiment

Twenty-three algal species from eight phyla were selected for the normal culture experiment (Table 1). They were cultured in replete nutrients and suitable conditions by the Freshwater Algae Culture Collection of the Institute of Hydrobiology, Chinese Academy of Sciences. The media are listed in Supplementary Table 1, and their detailed components can be found on the website:^1^ Algae were collected at their exponential phases. The photosynthetic parameters were measured by a Phyto-PAM fluorometer (Phyto-PAM, ED, Walz, Germany) with Phyto-Win software (Version 2.11), and other relevant physiological parameters of algae were analyzed by CytoSense (CytoBuoy b.v., the Netherlands).

**Table 1:** Physiological parameters for 23 algal species at the exponential growth phase in normal culture experiment in this study.

Algal species	Cell volume (μm^3^)	Abundance (cells mL^−1^)	Carbon content (pg cell^−1^)	*F_v_*/*F*_*m*_
**Chlorophyta**				
*Chlorella pyrenoidosa* FACHB-9	65.4	1.50 × 10^6^	10.96	0.69
*Ankistrodesmus* sp. FACHB-47	973.0	1.22 × 10^5^	135.27	0.70
*Closterium sp.* FACHB-61	141962.3	9.59 × 10^3^	14932.75	0.73
*Scenedesmus bijuga* FACHB-76	894.6	1.54 × 10^5^	129.12	0.69
*Chlamydomonas reinhardtii* FACHB-479	320.0	6.47 × 10^5^	47.27	0.74
*Haematococcus pluvialis* FACHB-872	9670.3	1.02 × 10^4^	1190.12	0.66
*Protococcus viridis* FACHB-891	97.6	2.15 × 10^5^	16.65	0.73
*Oocystis sp.* FACHB-1425	852.4	3.94 × 10^4^	123.15	0.68
**Cyanophyta**				
*Nostoc sp.* FACHB-106	60.1	3.97 × 10^4^	9.77	0.56
*Anabaena cylindrica* FACHB-170	39.8	2.91 × 10^4^	6.71	0.49
*Oscillatoria lutea* var.*contorta* FACHB-278	66.9	1.48 × 10^4^	10.96	0.44
*Microcystis aeruginosa* FACHB-315	36.4	6.12 × 10^4^	6.71	0.56
*Synechococcus elongatus* FACHB-347	227.5	8.98 × 10^4^	35.66	0.26
*Aphanizomenon flos-aquae* FACHB-1039	61.8	1.83 × 10^5^	10.96	0.34
*Pseudanabaena sp.* FACHB1277	34.3	4.22 × 10^4^	5.85	0.38
**Rhodophyta**				
*Porphyridium purpareum* FACHB-806	74.1	1.57 × 10^5^	12.24	0.40
*Rhodella reticulata* FACHB-807	90.7	7.91 × 10^5^	15.09	0.55
**Chrysophyta**				
*Prymnesium parvum* FACHB-967	79.6	1.27 × 10^6^	13.62	0.64
*Isochrysis galbana* FACHB-1123	82.4	1.26 × 10^6^	13.62	0.57
**Pyrrophyta**				
*Peridinium umbonatum var. inaequale* FACHB-329	21026.2	1.18 × 10^4^	2467.66	0.45
**Euglenophyta**				
*Euglena gracilis* FACHB-277	4501.9	2.14 × 10^5^	575.65	0.54
**Bacillariophyta**				
*Stephanodiscus sp.* FACHB-986	1090.7	1.24 × 10^5^	84.10	0.66
**Cryptophyta**				
*Cryptomonas curvata* FACHB-1302	9646.1	2.58 × 10^4^	1190.12	0.67

In addition, *C. reinhardtii* was taken as a representative for culturing under different initial CO_2_ or nitrate concentrations. NaHCO_3_ and NaOH were used to adjust the initial CO_2_ concentrations to 3, 14, 52, 175, 497 and 804 μmol L^−1^, of which initial CO_2_ concentrations of 3 and 14 μmol L^−1^ were considered as CO_2_ limitation according to the saturation value of water CO_2_ under standard atmospheric pressure and present temperature (Li et al., 2022). NaNO_3_ was used as the only nitrogen source to adjust the initial nitrate concentrations of SE medium to 25, 50, 250, 500 and 2,941 μmol L^−1^, of which initial nitrates of 25 and 50 μmol L^−1^ were considered as nitrate limitation based on the decreased algal biomass and increased algal C:N ratio (Li et al., 2022). The detailed sampling and measurement methods of algal photosynthetic parameters, transcriptome sequencing, and nutrient concentrations of the medium are referred to Li et al. (2022).

### Study area and sampling

The phytoplankton assemblages and related environmental factors of the Pingzhai, Puding, and Yingzidu reservoirs on the Sancha River tributary of the Wujiang River, Southwest China, were investigated. Water samples were collected in July and October 2017 and January and April 2018 at different depths in different reservoirs (Supplementary Table 2). The Yuqiao (YQ), Chaobaihe (CBH), Bolonghu (BLH), Donglihu (DLH), Haihe (HH), Tuanbowa (TBW), and Yongdinghe (YDH) reservoirs and two inflowing waters from the CBH and TBW reservoirs (CBH1 and TBW1, respectively) were investigated in Tianjin, North China. Their surface water samples were collected in September 2020 (Supplementary Table 2). Detailed information about the sites is referred to Xiao et al. (2021) and Li et al. (2022).

In the field, water temperature, dissolved oxygen (DO), and pH were measured *in situ* with an automated multiparameter profiler (model YSI EXO, United States) with precorrection. The photosynthetic parameters of the samples were measured by the Phyto-PAM fluorometer within 12 h after sampling. 1.5 l water samples were kept still for more than 24 h and were concentrated to final volumes of 50 ml for the analyses of phytoplankton total abundance (TA) and biovolume. The particulate matter was collected by a Whatman GF/F membrane (450°C, 8 h) to measure the organic C content. Water samples were filtered by vacuum filter with 0.45 μm Millipore cellulose acetate membranes, and the filtered waters were prepared to measure nutrient concentrations. The sample for metatranscriptomic sequencing at each field site in Tianjin was collected by a 0.7 μm Whatman GF/F membrane and then stored in liquid nitrogen immediately.

### Physiological parameter measurement

Phytoplankton photosynthetic parameters, including chlorophyll *a* concentration (Chl *a*), maximum quantum yield (*F_v_*/*F_m_*), effective quantum yield (yield), light use efficiency (Alpha), maximum electron transfer rate (ETR_max_), and half-saturation light intensity (*I_k_*), were determined by the Phyto-PAM fluorometer. The sample was subjected to dark adaptation for 15 min. The minimal fluorescence *F_0_* was determined by turning on the measuring light, and the maximal fluorescence *F_m_* was obtained by the saturation light pulse of 4,000 μmol m^−2^ s^−1^. *F_v_*/*F_m_* was calculated, where *F_v_* was variable fluorescence equal to *F_m_*–*F_0_*. Without dark acclimation, the rapid light curve (RLC) was constructed by exposing the sample to 16, 32, 64, 164, 264, 364, 464, 564, 646 and 764 μmol m^−2^ s^−1^ actinic light, and the irradiation time per level was 20 s. Alpha is the initial slope of the RLC.

The fluorescence parameters of cultured algae were analyzed by the CytoSense. The instrument was equipped with a solid-state laser (488 nm, 15 mW) to detect the passing cells, and the forward scatter (FWS) was collected by a PIN photodiode. Sideward scatter (SWS), red fluorescence (668–734 nm, FLR), orange fluorescence (601–668 nm, FLO), and yellow fluorescence (536–601 nm, FLY) were separated by a concave holographic grating and collected on a hybrid photomultiplier. Data recording was triggered by FWS. The peristaltic pump controlled the flow rate within 80 ~ 120 μl min^−1^, and the injection time of each sample was approximately 5 min. Cytoclus software (CytoClus3, CytoBuoy, b.v., the Netherlands) was used to analyze the data measured by CytoSense. The fluorescence and size parameters of phytoplankton were determined by the amplitude and shape of multiple signals (FWS, SWS, FLR, FLO and FLY).

Algal species and number were determined by hemocytometer measurement using an Olympus CX31 microscope (Lund et al., 1958), and the biovolume of each species was geometrically calculated (Hillebrand et al., 1999). In the normal culture experiment, the cellular C contents were calculated by the following formulas (Verity et al., 1992; Menden-Deuer and Lessard, 2000):

log pg. C cell^−1^ = −0.665 + 0.939 (log V) (μm^3^) (excluding Bacillariophyta).

log pg. C cell^−1^ = −0.541 + 0.811 (log V) (μm^3^) (Bacillariophyta).

where the log base is 10, C is the cellular C content, and V is the cell volume.

In the field, the mean cell volume of the phytoplankton assemblage (Vol) was calculated by the following formula:

Vol$=\left(\overset{n}{\underset{i=1}{\sum }}V_{i}A_{i}\right)$ / $\left(\overset{n}{\underset{i=1}{\sum }}A_{i}\right).$where *V_i_* is the cell volume of the *i*th species, *A_i_* is the abundance of the *i*th species, and *n* is the number of species encountered.

The Shannon–Wiener index (H՛) of phytoplankton was calculated according to the equation:


$$\mathrm{H՛}=-\overset{n}{\underset{i=1}{\sum }}P_{i}\;\left(lnP_{i}\right)$$

where P*_i_* is the ratio of *i*th species number to total number, and *n* is the number of species encountered.

The carbon density was the ratio of cellular carbon content to cellular volume for the single species in the laboratory or mean carbon content to mean biovolume for the phytoplankton assemblage in the field, whereas the Chl *a* density was the ratio of Chl *a* concentration to total biovolume of either single species in the laboratory or phytoplankton assemblage in the field. The other relevant parameters, including water alkalinity (Alk), dissolved inorganic nitrogen (DIN, sum of ammonium nitrogen, nitrate nitrogen, and nitrite nitrogen), phosphate phosphorus (PO_4_^3−^-P), dissolved silicon (DSi), and particulate organic carbon (POC), were also determined, and the detailed analytical methods were referred to Xiao et al. (2021) and Li et al. (2022).

### Transcriptome and metatranscriptomic sequencing

Transcriptome and metatranscriptomic sequencing were conducted by commercial services (Biomarker Technologies Co., Ltd., Beijing, China and Allwegene Technologies Co., Ltd., Beijing, China). The raw data of transcriptome sequencing were deposited in the NCBI SRA database with accession number PRJNA688010, and those of metatranscriptomic sequencing were deposited in the NCBI SRA database with accession number PRJNA798457.

Clean reads were obtained from raw reads by removing reads containing adapters and low-quality reads. All downstream analyses were based on high-quality clean reads. Gene expression levels were estimated by fragments per kilobase of transcription per million fragments mapped (FPKM). In transcriptome analyses, fold change (FC) represents the ratio of FPKMs between the nutrient limitation group and the normal control group. The *p* value was calculated using Benjamini and Hochberg’s approach for controlling the false discovery rate (FDR), and this rate was the key index of differentially expressed gene (DEG) screening. |Log_2_ FC| ≥ 1 and FDR < 0.01 were used as criteria for screening genes for further analyses, and |Log_2_ FC| < 1 and FDR > 0.01 were considered as insignificant difference in gene expression levels between the different groups. In metatranscriptome analyses, average gene expression levels (AGELs) of proteins involved in PS II, energy synthesis (i.e., the synthesis of ATP and NADPH in photosynthesis), carbon fixation, cytoskeleton, and cell wall were calculated from their respective FPKMs. Volume-specific AGEL was the ratio of AGEL in a certain pathway to Vol.

### Data collection and analysis

The cell volume and *F_v_*/*F_m_* of different algal species in the exponential growth phase under normal culture conditions were obtained from the published literature, and for those studies without cell volumes, they were calculated according to Hillebrand et al. (1999). The details are presented in Supplementary Table 3. The size of cell volume was divided into “small” (<10^2^ μm^3^), “intermediate” (10^2^–10^4^ μm^3^), and “large” (>10^4^ μm^3^) (Maranon et al., 2013; Maranon, 2015; Montes-Pérez et al., 2020).

Data plotting were conducted using Origin2017. One-way analysis of variance (ANOVA) and T-test were used to determine the significant differences within the 95% confidence interval between different groups using IBM SPSS statistics 24. Pearson correlation analysis was conducted by R software (version 3.4.2).

## Results

### Normal culture experiment

**Figure 1:**
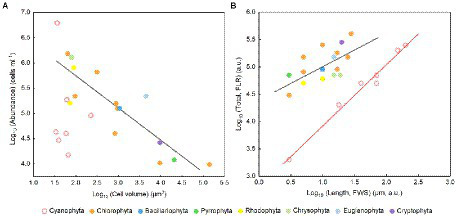
**(A)** Algal cell volume versus abundance in the normal culture experiment. The regression for algae except Cyanophyta: *y* = −0.64*x* + 7.02, adjusted *r*^2^ = 0.74, *p* < 0.01. **(B)** FLR area per cell versus FWS length per cell. FLR, red fluorescence; FWS, forward scatter. The regression for eukaryotic algae (black line): *y* = 0.59*x* + 4.43, adjusted *r*^2^ = 0.35*, p* < 0.01; the regression for Cyanophyta (red line): *y* = 1.12*x* + 2.80, adjusted *r*^2^ = 0.98, *p* < 0.001.

**Figure 2:**
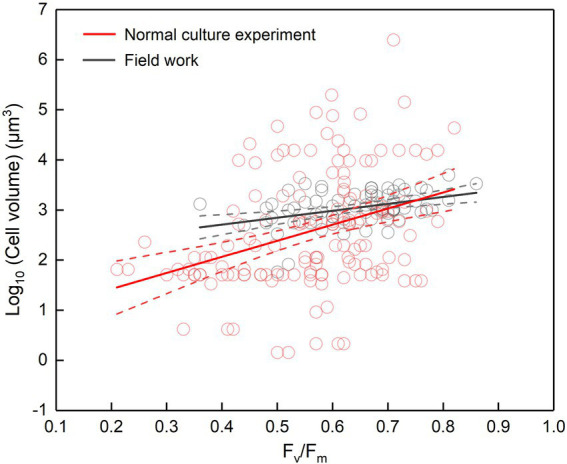
*F_v_*/*F_m_* versus cell volume. Cell volume is either the cell volume of certain species in the normal culture experiment (including the collected data) or the mean cell volume of phytoplankton assemblage in the field work. The regression for normal culture experiment: *y* = 3.21*x* + 0.77, adjusted *r*^2^ = 0.12, *p* < 0.001; the regression for field work: *y* = 1.38*x* + 2.15, adjusted *r*^2^ = 0.15, *p* < 0.001; the dotted lines represent the 95% confidence intervals.

**Figure 3:**
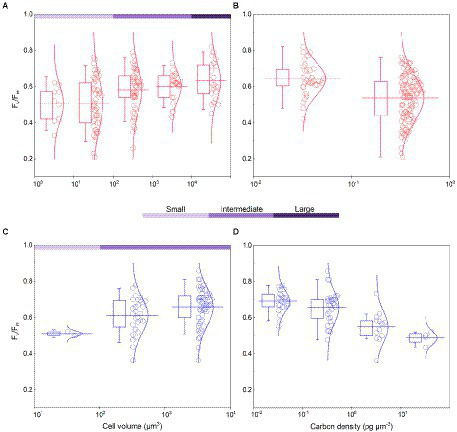
Algal *F*_*v*_/*F*_*m*_ and carbon density in the normal culture experiment [**(A,B)**, including the collected data] and field work **(C,D)**. The classification of cell volume is referred to the text, and carbon density is nominally distributed on a scale of 10. The curve represents the normal distribution of data. The box encompasses the 25–75th percentiles, and whiskers and midline are the standard deviation and mean, respectively.

The cell abundance in the exponential growth phase of 23 algal species ranged from 10^3^ to 10^7^ cells ml^−1^, with an average of 5.7 × 10^5^ cells ml^−1^, and the cell volume ranged from 10 to 10^5^ μm^3^, with an average of 8.3 × 10^3^ μm^3^. There was a significant logarithmic negative correlation between cell abundance and cell volume for the eukaryotic algae (Figure 1A). The calculated C density ranged from 0.08 to 0.17 pg. μm^−3^, with an average of 0.15 pg. μm^−3^. Algal *F_v_*/*F_m_* showed an average of 0.57 and displayed obvious species specificity. The maximum was 0.74 for *C. reinhardtii*, and the minimum was 0.26 for *Synechococcus elongatus* (Table 1). There was small differentiated *F_v_*/*F_m_* among the eukaryotic algae, but these *F_v_*/*F_m_* was significantly different from that of the Cyanophyta (T-test, *p* < 0.001; Table 2). In addition, *F_v_*/*F_m_* was significantly positively correlated, respectively, with yield (*r* = 0.54, *p* < 0.01), Alpha (*r* = 0.86, *p* < 0.01), and ETR_max_ (*r* = 0.47, *p* < 0.05), indicating it can be a representative among photosynthetic parameters. The FLR (representing the content of chlorophyll) and FWS (representing the cell size) were species specific, and two clusters were found as prokaryotic algae (i.e., Cyanophyta) and eukaryotic algae in Figure 1B. The FLR of Cyanophyta was notably lower than that of eukaryotic algae, and the FLR was significantly positively correlated with FWS either among eukaryotic algae or within Cyanophyta (Figure 1B). In addition, the *F_v_*/*F_m_* from published literature of different algal phyla showed similar value to that of the culture experiment (T-test, *p* = 0.426; Table 2). The integrated *F_v_*/*F_m_* from published data and culture experiment was positively logarithmically correlated with Vol and negatively logarithmically correlated with carbon density (Figure 2; Table 3). The increasing trend of *F_v_*/*F_m_* became less apparent when the cells were intermediate and large (Figure 3A).

**Table 2:** *F_v_*/*F_m_* of different algal phyla.

Phylum	*F_v_*/*F_m_*^a^	*F_v_*/*F_m_*^b^
Cyanophyta	0.42 ± 0.10	0.43 ± 0.11
Chlorophyta	0.56 ± 0.17	0.70 ± 0.03
Chrysophyta	0.62 ± 0.05	0.61 ± 0.05
Cryptophyta	0.58 ± 0.03	0.67
Bacillariophyta	0.63 ± 0.09	0.66
Pyrrophyta	0.54 ± 0.07	0.45
Phaeophyta	0.50 ± 0.05	/
Rhodophyta	/	0.48 ± 0.11
Euglenophyta	/	0.54

Values are presented as mean ± standard deviation.

a , from the published literature (the details referred to Supplementary Table 3).

b , from the cultured algae (the details referred to Table 1).

/, no data available.

**Table 3:** Linear regression of *F_v_*/*F_m_* and each Log_10_ transformed physiological parameter (i.e., cell volume, C density, and Chl *a* density).

*x*	*a*	*b*	Adjusted *r*^2^	*p*	*n*
Cell volume	0.04 ± 0.01	0.46 ± 0.02	0.12	<0.001	145^A^
	0.12 ± 0.03	0.29 ± 0.10	0.15	<0.01	69^C^
C density	−0.24 ± 0.05	0.34 ± 0.04	0.15	<0.001	145^A^
	−0.08 ± 0.01	0.60 ± 0.01	0.41	<0.001	69^C^
Chl *a* density	−0.06 ± 0.02	0.60 ± 0.03	0.22	<0.05	23^B^
	−0.08 ± 0.02	0.67 ± 0.02	0.16	<0.001	69^C^

The regression equation: *F_v_*/*F_m_* = *a* Log_10_ *x* + *b*, where *x* is the independent variable, *a* is the slope, and *b* is the *y*-intercept; *a* and *b* are shown as mean ± standard deviation; adjusted *r*^2^, the adjusted square of correlation coefficient; *p*, *p* value; *n*, the number of data; superscripts, different data groups.

A , culture experiment and collection data.

B , culture experiment.

C , field work.

### Conditional culture experiment for *Chlamydomonas reinhardtii*

**Figure 4:**
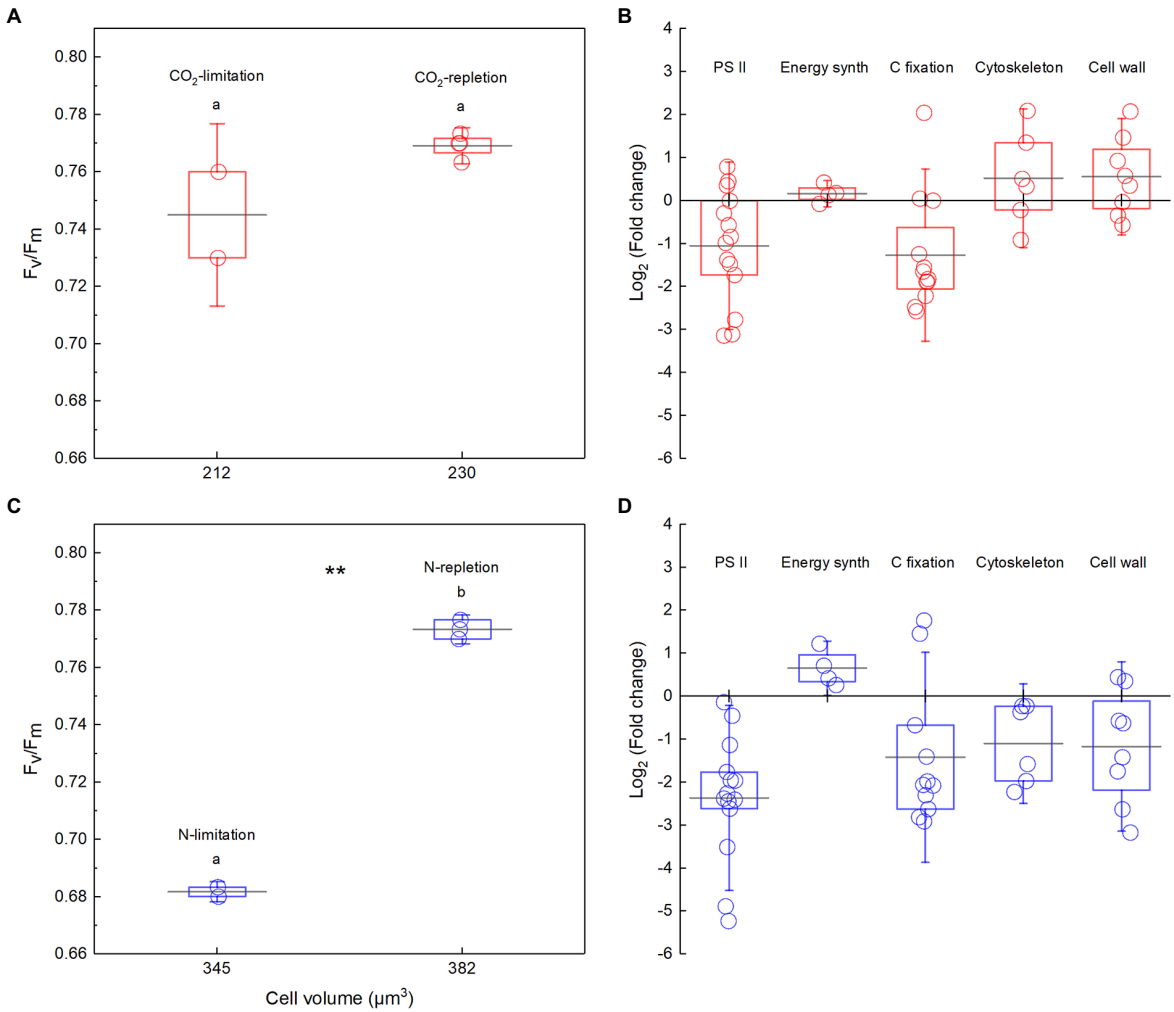
**(A)** *F*_*v*_/*F*_*m*_ and cell volume of *Chlamydomonas reinhardtii* under different CO_2_ concentration conditions. **(B)** The fold change of gene expression of proteins involved in Photosystem II (PS II), energy synthesis (energy synth), carbon fixation (C fixation), cytoskeleton, and cell wall under CO_2_ limitation in comparison with CO_2_ repletion. **(C)** *F*_*v*_/*F*_*m*_ and cell volume of *Chlamydomonas reinhardtii* under different nitrate concentration conditions. **(D)** The fold change of gene expression of proteins involved in PS II, energy synth, C fixation, cytoskeleton, and cell wall under nitrate limitation in comparison with nitrate repletion. The box encompasses the 25th-75th percentiles, and whiskers and midline are the standard deviation and mean, respectively. The lowercase letters represent the significance of the difference in *F_v_*/*F_m_* (*p* < 0.05) between different conditions. **represents the significant difference in cell volume at the 0.01 level between different conditions.

In the culture experiment for *C. reinhardtii*, *F_v_*/*F_m_* ranged from 0.73 to 0.77, with an average of 0.76, and cell volume ranged from 204 to 248 μm^3^, with an average of 221 μm^3^, in the CO_2_-gradient experiments. Compared with the CO_2_-replete condition, cell volume and *F_v_*/*F_m_* were not significantly lower under CO_2_ limitation (Figure 4A); the average expression levels of proteins involved in PS II, energy synthesis, cytoskeleton, and cell wall also showed insignificant change, whereas that of carbon fixation was significant down-regulated (Figure 4B; Supplementary Table 4). The Log_2_ FC of proteins involved in PS II was −1.05, and its average expression level was thus considered as an insignificant downregulation in this study. In the nitrate gradient experiments, *F_v_*/*F_m_* ranged from 0.68 to 0.78, with an average of 0.74, and cell volume ranged from 321 to 421 μm^3^, with an average of 369 μm^3^. Compared with the nitrate-replete condition, both cell volume and *F_v_*/*F_m_* were significantly lower under nitrate limitation (Figure 4C); average expression levels of proteins involved in PS II, carbon fixation, cytoskeleton, and cell wall were significantly downregulated, but that of energy synthesis was not (Figure 4D; Supplementary Table 5).

### Field work

**Figure 5:**
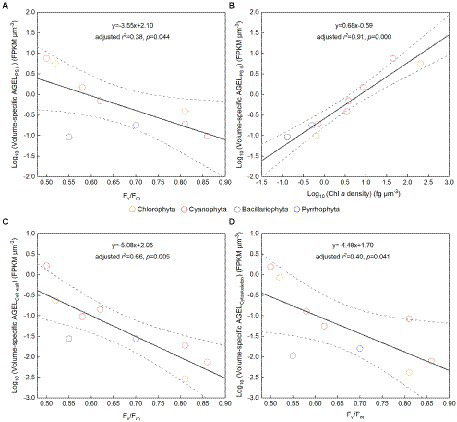
**(A)** *F*_*v*_/*F*_*m*_ versus volume-specific average gene expression level (AGEL) of Photosystem II (PS II) proteins; **(B)** Chlorophyll *a* (Chl *a*) density versus volume-specific AGEL of PS II proteins; **(C)** *F*_*v*_/*F*_*m*_ versus volume-specific AGEL of proteins involved in cell wall; **(D)** *F*_*v*_/*F*_*m*_ versus volume-specific AGEL of proteins involved in cytoskeleton; all for the phytoplankton assemblage in Tianjin’s reservoirs. Different color point represents different dominant algal group in each sampling site. The dotted lines represent the 95% confidence intervals.

**Figure 6:**
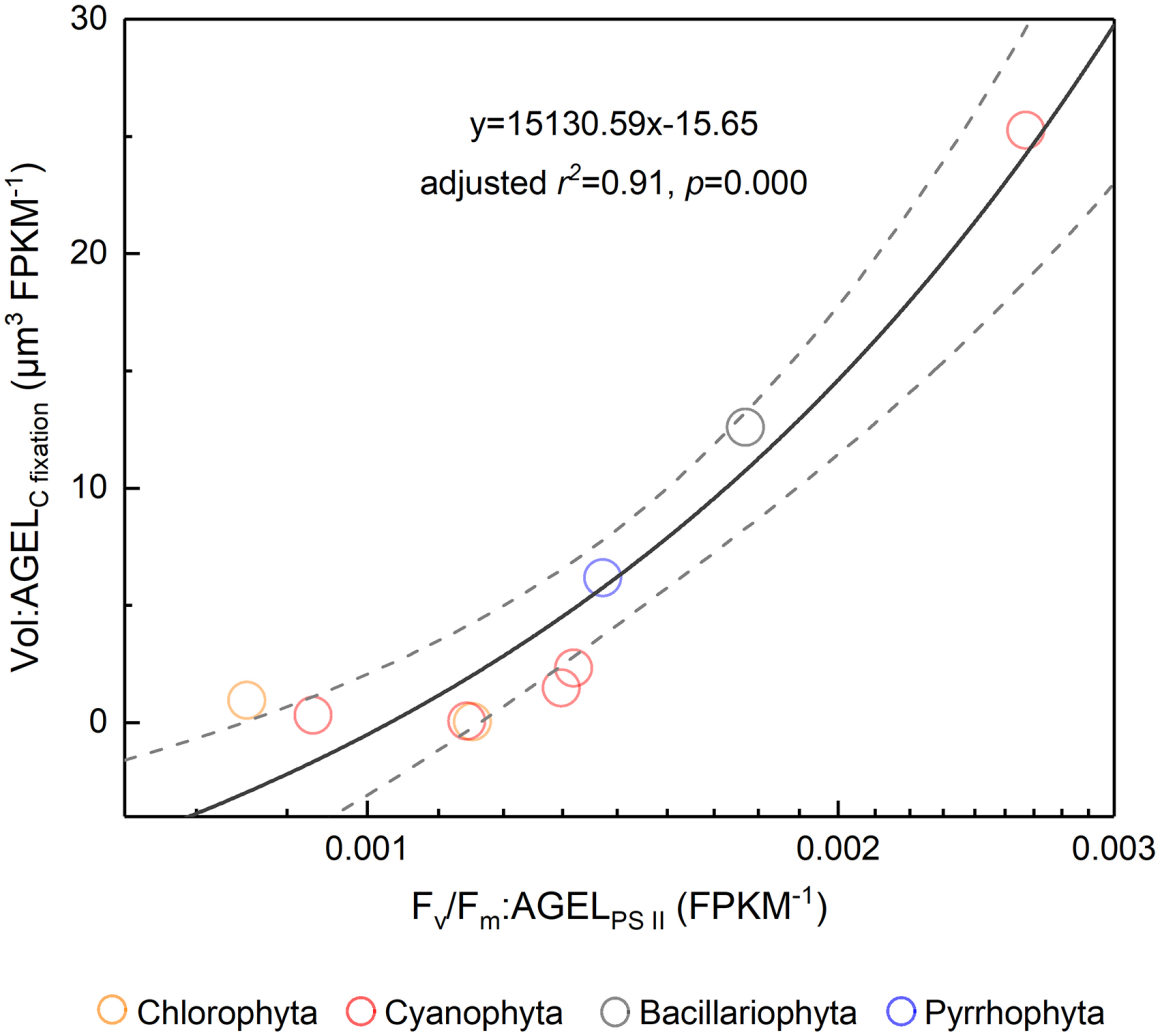
The ratio of *F_v_*/*F_m_* to PS II protein AGEL versus the ratio of Vol to C-fixed protein AGEL for the phytoplankton assemblage in Tianjin’s reservoirs. PS II, Photosystem II; C, carbon; AGEL, average gene expression level; Vol, mean cell volume of phytoplankton assemblage. Different color point represents different dominant algal group in each sampling site. The dotted lines represent the 95% confidence intervals.

The *F_v_*/*F_m_* of phytoplankton assemblage ranged from 0.36 to 0.86, with an average of 0.66. The *F_v_*/*F_m_* was significantly positively correlated with TA (*p* < 0.01) and negatively correlated with H՛ (*p* < 0.05; Supplementary Figure 1). The Vol ranged from 58 to 6,879 μm^3^, with an average of 1,316 μm^3^, dominated by small and intermediate cells (Figure 3C). The Vol was significantly positively correlated with Alpha, Chl *a*, DIN, and DO (Supplementary Figure 1). The *F_v_*/*F_m_* was significantly positively correlated with Vol and negatively correlated either with C density or Chl *a* density (Figure 2; Table 3). In the metatranscriptome analyses, the maximum AGEL of proteins involved in PS II was 967 in YDH, and the minimum was 315 in YQ (Supplementary Table 6). The maximum AGEL of energy synthesis was 500 in TBW1, and the minimum was 50 in CBH (Supplementary Table 6). For the AGEL of C fixation, the maximum was 5,274 in YDH, and the minimum was 133 in CBH (Supplementary Table 6). The AGELs of proteins involved in PS II, energy synthesis, and C fixation were significantly positively correlated with each other (Supplementary Figure 2). For the AGELs of cytoskeleton and cell wall, they showed obvious different among the sampling sites (Supplementary Table 6). The *F_v_*/*F_m_* was negatively correlated with volume-specific AGELs of proteins involved in PS II, cytoskeleton, and cell wall, respectively (Figure 5; Table 3). The Chl *a* density was positively correlated with carbon density and volume-specific AGEL of PS II proteins, respectively (Figure 5B and Supplementary Figure 3). The ratio of *F_v_*/*F_m_* to PS II protein AGEL had a significant relationship with the ratio of Vol to carbon-fixation AGEL, independent of phytoplankton community structure (Figure 6 and Supplementary Figure 4).

## Discussion

### Relationship between *F_v_*/*F_m_* and cell size for phytoplankton

It seems to be universal that *F_v_*/*F_m_* has a positive relationship with cell size although the slope of regression curve was different between the laboratory and the field (Figure 2). In the normal culture experiment, *F_v_*/*F_m_* and cell size can exhibit the phytoplankton genetic performance with their growth freeing from environmental stress and species competition. The integrated data of *F_v_*/*F_m_* included a large number of algal species, and thus the corresponding cell size ranged widely. However, in the field, the number of dominant species was much smaller than that of all integrated algal species, and thus the cell size had a narrower distribution. In addition, the evolving larger cell of *Dunaliella teriolecta* by an artificial selection displayed higher *F_v_*/*F_m_* in a previous experimental study (Malerba et al., 2018), and in the conditional culture experiment, cell size of *C. reinhardtii* decreased with the decreasing *F_v_*/*F_m_* under nitrate limitation (Figure 4C).

*F_v_*/*F_m_* is the quantum yield when all active centers of light-harvesting system PS II are open (Consalvey et al., 2005) and is thus directly determined by the types of pigment-protein complexes in PS II (Stanier and Cohen-Bazire, 1977). The differences in structure and amounts of pigment-protein complexes could be the primary cause leading to a large difference in *F_v_*/*F_m_* between cyanobacteria and eukaryotic algae. For example, Cyanophyta has a phycobilisome allowing absorption and unidirectional transfer of light to chlorophyll *a*, whereas Chlorophyta and Euglenophyta have complexes combining chlorophyll *a* and *b* (Grossman et al., 1995). In addition, the chlorophyll content of Cyanophyta was notably lower than that of eukaryotic algae (Figure 1B), and the phycobiliproteins of cyanobacteria contributing fluorescence overlap with the spectrum of PS II chlorophyll emission (Campbell et al., 1998) and enhance the minimal fluorescence yield F_0_, finally reducing the *F_v_*/*F_m_* of cyanobacteria. The differences in *F_v_*/*F_m_* among eukaryotic algae are probably mainly due to the differences in amounts rather than structure of pigment-protein complexes. In the field, the variation in *F_v_*/*F_m_* of phytoplankton assemblage could be attributed to the different phytoplankton community structure among the reservoirs (Supplementary Figure 1). It has reported that taxonomic diversity of phytoplankton is a function of cell size (Ryabov et al., 2021), implying that there should be a link between *F_v_*/*F_m_* and cell size among the interspecies.

For the relation of *F_v_*/*F_m_* to cell size within the intraspecies, the package effect could give a theoretical explanation. The package effect is that the absorption of pigments in cells decreases in comparison with the absorption potential for same amount of pigment in solution (Raven, 1984; Stuart et al., 1998). For microalga *Dunaliella teriolecta*, volume-specific photosynthetic pigments has been reported to increase with an increase in cell size (Malerba et al., 2018), as large cells might reduce the influence of increasing cellular package effect by improving photosynthetic performance.

Environmental factors can influence phytoplankton *F_v_*/*F_m_* and cell size. This study demonstrated that nitrate limitation resulted in a significant decrease of *F_v_*/*F_m_* and cell size (Figure 4C), and other studies also reported that nutrient availability and warming can trigger a change of phytoplankton cell size (Maranon et al., 2013; Hillebrand et al., 2022a,b). In addition, environmental stress such as ultraviolet radiation and heavy metals can affect *F_v_*/*F_m_* by damaging the structure and metabolism of pigment-protein complexes in phytoplankton cells (Kolber et al., 1994; Beecraft et al., 2019), and in a response, the *F_v_*/*F_m_* and cell size are both changed (Tan et al., 2019; Hillebrand et al., 2022a).

In a word, the difference in light-harvesting systems among the interspecies, the cellular package effect, the nutrient availability, water temperature, and other environmental stresses can cause the difference in photosynthetic light energy utilization for phytoplankton, which could ultimately affect phytoplankton cell size through feedback of cell metabolism to current ambient changes or long-term evolution. Therefore, phytoplankton *F_v_*/*F_m_* positively correlates with cell size, and it is highlighted on the intrinsic cause relationship between photosynthetic light energy utilization and phytoplankton cell size control in this study.

### Molecular mechanisms regulating cell size by photosynthetic light energy utilization for phytoplankton

**Figure 7:**
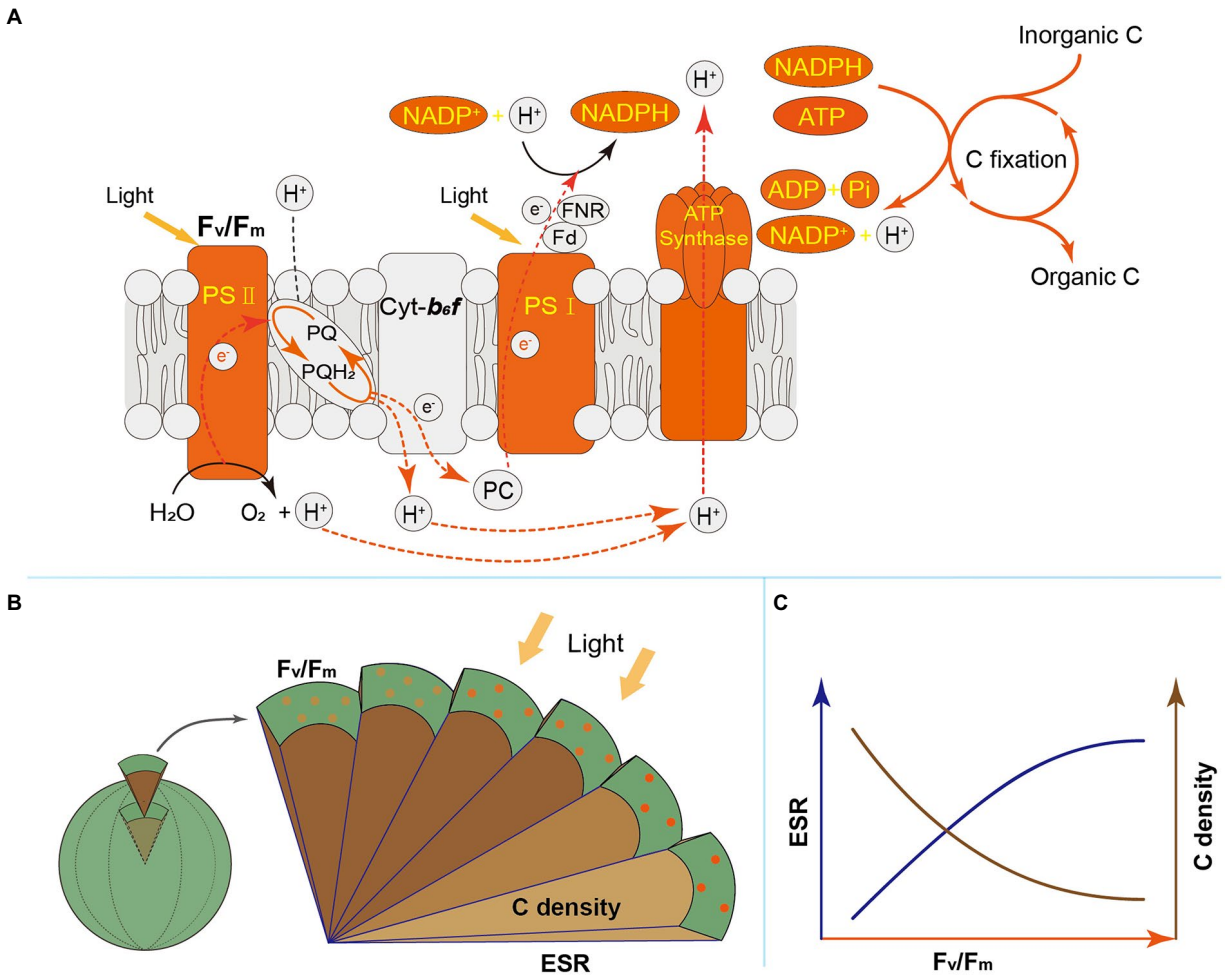
**(A)** Schematic diagram for photosynthesis and carbon fixation in phytoplankton cells. The orange marks represent the regulation pathways that is highlighted in this study. **(B)** Schematic diagram for regulation of light energy utilization on cell size of phytoplankton. The circles on the surface: the chlorophyll molecules. *F_v_*/*F_m_*: the maximum quantum yield of Photosystem II, marked by orange; ESR: equivalent spherical radius, marked by dark blue; C density: carbon density, marked by brown. A gradual increase in color indicates higher values of F_v_/F_m_ and C density; with the increasing ESR, F_v_/F_m_ increases, but C density decreases. **(C)** Schematic diagram for the relationship between F_v_/F_m_ and ESR (or C density) for phytoplankton interspecies.

Light energy is a prerequisite for the survival of phytoplankton. In the cellular thylakoid membrane, PS II harvests light and produces electrons and H^+^, which drive the formation of ATP and NADPH that release to the stroma for carbon fixation (Figure 7A). A part of fixed carbon is used to structure cytoskeleton and cell wall (or cell wall-like such as a glycocalyx), and the rest enters the other metabolism such as lipid synthesis. Based on the metatranscriptome analyses from Tianjin’s reservoirs, for the proteins involved in PS II, energy synthesis, and carbon fixation, their gene expression changed synchronously, accompanying with the varying cell size among phytoplankton interspecies (Supplementary Figure 2). Therefore, the molecular evidence supported that there will be a relationship between *F_v_*/*F_m_* and cell size. Cellular gene expression responds to the present environmental variations including nutrient limitation and/or species competition (Hockin et al., 2012; Narwani et al., 2017). For phytoplankton assemblage, the ratio of *F_v_*/*F_m_* to PS II protein AGEL is specific *F_v_*/*F_m_* normalized by AGEL of PS II proteins under different environmental conditions, and the ratio of biovolume to carbon-fixed protein AGEL is also a similar meaning. They were significantly correlated each other (Figure 6), verifying that there is a universal relationship between light energy utilization and cell size, and phytoplankton cells are the unity of light energy conversion and matter metabolism.

Nitrate limitation can substantially decrease the synthesis of nitrogenous macromolecules such as pigment-protein complexes (Osborne and Geider, 1986; Mock and Kroon, 2002), and the gene expression of PS II proteins was thus significant downregulated, causing a decrease of *F_v_*/*F_m_* in the conditional experiment (Figures 4C,D). This effect was passed on the gene expression of the proteins involved in carbon fixation, cytoskeleton, and cell wall in turn, finally resulting in a decrease of cell size (Figures 4C,D). CO_2_ limitation showed less impact on *C. reinhardtii* than nitrate limitation because the cell operated CO_2_-concentrating mechanisms to offset the lack of CO_2_ (Wang et al., 2013; Maberly and Gontero, 2017; Li et al., 2022). As such, gene expression of carbon-fixed proteins was significant downregulated (Figure 4B) because CO_2_-concentrating mechanisms are energy-dependent and compete for the energy (e.g., ATP) required for carbon fixation (Wang et al., 2015). However, algal light energy utilization was not seriously affected, neither did the cellular gene expression of proteins involved in PS II, cytoskeleton, and cell wall (Figure 4B). Accordingly, there was no significant change in cell size. All the molecular evidence demonstrated that photosynthetic light energy utilization regulates phytoplankton cell size by harmonizing the generation and allocation of cellular chemical energy and fixed carbon in the cell. This finding not only provides an explanation on that environmental stress (e.g., nutrient limitation) influencing phytoplankton light conversion can usually decrease their cell size (Kolber et al., 1994; Li et al., 2022) but also interprets why phytoplankton cell size can be an important functional trait.

Accompanying with the formation of species diversity, phytoplankton evolve their own cell size diversity (Acevedo-Trejos et al., 2018). However, in the long-term evolution, phytoplankton could not enlarge their cell size infinitely. The first evidence is a decrease of volume-specific Chl *a* with an increase in cell volume for phytoplankton interspecies (Figure 7B; Tetsuichi and Satoru, 2002). In this study, the nonlinear relationship between *F_v_*/*F_m_* and cell size (Figure 2) and the nonlinear decrease of volume-specific PS II protein AGEL with the increase in *F_v_*/*F_m_* supported that with the increasing *F_v_*/*F_m_*, the increase in cell size was fast when *F_v_*/*F_m_* was small but became slow when *F_v_*/*F_m_* was large (Figures 5A, 7C). In addition, the volume-specific carbon (i.e., carbon density) decreased with the increasing *F_v_*/*F_m_* (Figures 3B,D, 7C), implying that the contribution of nascent fixed carbon will decrease with an increase in cell size. The volume-specific AGELs of proteins involved in cytoskeleton and cell wall showed similar behaviors (Figures 5C,D), indicating that phytoplankton cells may sense their own growth and give negative feedback to persistent cell enlargement. Therefore, it can be inferred that phytoplankton cell size ceases to enlarge once the increased light energy conversion and subsequent fixed carbon could no longer satisfy the increasing demand of volume enlargement.

Phytoplankton cell size, as an important functional trait, is closely related to their *F_v_*/*F_m_*, and this relationship was usually considered as the results of the former constraining the latter. Here, we interpret this relationship from a reverse angle and regard the utilization of photosynthetic light energy as the reason for the size control, based on the fact that phytoplankton are the unity of material and energy, but the acquisition of light energy is the premise of their survival. Phytoplankton cell size, to some degree, is the results of cell responding to ambient changes and then balancing internal metabolism. Many environmental factors can shape phytoplankton cell size by influencing the utilization of light energy. It should be noted, however, that cell size is a relatively robust variable, while *F_v_*/*F_m_* is much more active, with relatively large variation and fast response to environmental changes that can work on a time scale of seconds to hours. Therefore, when analyzing the relationship from the perspective of using photosynthetic light energy as the cause of cell size, the *F_v_*/*F_m_* had better be determined after the total adaptation to ambient conditions for phytoplankton cells, whether at normal conditions or at environmental stresses.

## Conclusion

There was a universal significant positive relationship between *F_v_*/*F_m_* and cell volume in general. The molecular evidence demonstrated that photosynthetic light energy utilization regulates phytoplankton cell size by harmonizing the generation and allocation of chemical energy and fixed carbon in the cell. Phytoplankton cell size would cease to enlarge once the increased light energy conversion and subsequent fixed carbon could no longer satisfy the increasing demand of size enlargement. Cell size control of phytoplankton is a complex process, and more elaborate regulation mechanisms are worthy of further studies for refreshing our knowledge.

## Data availability statement

The data presented in the study are deposited in the NCBI SRA repository, accession number PRJNA688010 and PRJNA798457.

## Author contributions

BW developed the idea. BW, SX, and C-QL optimized study. WL, MY, and JX performed the experiment and field work. WL and BW produced the first draft. All authors contributed to the article and approved the submitted version.

## Funding

This work was financially supported by the National Natural Science Foundation of China (U1612441) and the National Key Research and Development Program of China (2016YFA0601001).

## Conflict of interest

The authors declare that the research was conducted in the absence of any commercial or financial relationships that could be construed as a potential conflict of interest.

## Publisher’s note

All claims expressed in this article are solely those of the authors and do not necessarily represent those of their affiliated organizations, or those of the publisher, the editors and the reviewers. Any product that may be evaluated in this article, or claim that may be made by its manufacturer, is not guaranteed or endorsed by the publisher.

## Footnotes

^1^ http://algae.ihb.ac.cn/MeSearch.aspx
