# Supplementary material for: Phytoplankton cell size control can be affected by photosynthetic light energy utilization
Source: Front Microbiol. 2022 Nov 2;13:1008606. doi: 10.3389/fmicb.2022.1008606 (PMC9667819; doi:10.3389/fmicb.2022.1008606)
Supplement: Supplementary file 1 [file Data_Sheet_1.docx]

# Supporting information

# (Table S1-S6; Figure S1-S4)

### Phytoplankton cell size control can be affected by photosynthetic light energy utilization

Wanzhu Li, Baoli Wang*, Jing Xiao, Meiling Yang, Sheng Xu, Cong-Qiang Liu Institute of Surface-Earth System Science, School of Earth System Science, Tianjin University, Tianjin 300072, China

***Corresponding author:** Baoli Wang (Email: baoli.wang@tju.edu.cn), Institute of Surface-Earth System Science, School of Earth System Science, Tianjin University, Tianjin 300072, China. Tel: +86 (0)22 27405053; Fax: +86 (0)22 27405051.

**Table**

**Table S1**. Algae species and medium in the culture experiment.

| Algal species | Medium | Algal species | Medium |
| --- | --- | --- | --- |
| *Chlorella pyrenoidosa* FACHB-9 | SE | *Haematococcus pluvialis* FACHB-872 | BG11 |
| *Ankistrodesmus* sp. FACHB-47 | SE | *Protococcus viridis* FACHB-891 | BG11 |
| *Closterium* sp. FACHB-61 | SE | *Oocystis* sp*.* FACHB-1425 | BG11 |
| *Scenedesmus bijuga* FACHB-76 | SE | *Nostoc* sp*.* FACHB-106 | BG11 |
| *Chlamydomonas reinhardtii* FACHB-479 | SE | *Anabaena cylindrica* FACHB-170 | BG11 |
| *Synechococcus elongatus* FACHB-347 | SE | *Oscillatoria lutea var.contorta* FACHB-278 | BG11 |
| *Porphyridium purpareum* FACHB-806 | KOCK | *Microcystis aeruginosa* FACHB-315 | BG11 |
| *Rhodella reticulata* FACHB-807 | KOCK | *Aphanizomenon flos-aquae* FACHB-1039 | BG11 |
| *Prymnesium parvum* FACHB-967 | Erdschreiber | *Pseudanabaena* sp*.* FACHB-1277 | BG11 |
| *Isochrysis galbana* FACHB-1123 | Erdschreiber | *Euglena gracilis* FACHB-277 | HUT |
| *Stephanodiscus* sp*.* FACHB-986 | CSI | *Cryptomonas curvata* FACHB-1302 | AF-6 |
| *Peridinium umbonatum var. inaequale* FACHB-329 | 119 |  |  |

**Table S2**. The sampling time and depth in the field work.

| Date | Reservoirs | Site | Site type | Depth |
| --- | --- | --- | --- | --- |
| 2017-July | Pingzhai | S1 | River | Surface |
|  |  | S2 | Reservoir | Surface |
|  |  |  |  | 5m |
|  |  |  |  | 15m |
|  |  | S3 | River | Surface |
|  | Puding | S4 | River | Surface |
|  |  | S5 | River | Surface |
|  |  | S6 | River | Surface |
|  |  | S7 | Reservoir | Surface |
|  |  |  |  | 5m |
|  |  |  |  | 10m |
|  |  |  |  | 15m |
|  |  | S8 | River | Surface |
|  | Yingzidu | S9 | Reservoir | Surface |
|  |  |  |  | 5m |
|  |  |  |  | 15m |
|  |  | S10 | River | Surface |
| 2017-October | Pingzhai | S1 | River | Surface |
|  |  | S2 | Reservoir | Surface |
|  |  |  |  | 5m |
|  |  |  |  | 15m |
|  |  | S3 | River | Surface |
|  | Puding | S4 | River | Surface |
|  |  | S5 | River | Surface |
|  |  | S6 | River | Surface |
|  |  | S7 | Reservoir | Surface |
|  |  |  |  | 5m |
|  |  |  |  | 10m |
|  |  |  |  | 15m |
|  |  | S8 | River | Surface |
|  | Yingzidu | S9 | Reservoir | Surface |
|  |  |  |  | 5m |
|  |  |  |  | 10m |
|  |  |  |  | 15m |
|  |  | S10 | River | Surface |
| 2018-January | Pingzhai | S1 | River | Surface |
|  |  | S2 | Reservoir | Surface |
|  |  |  |  | 5m |
|  |  |  |  | 15m |
|  |  | S3 | River | Surface |
|  | Puding | S4 | River | Surface |
|  |  | S5 | River | Surface |
|  |  | S6 | River | Surface |
|  |  | S7 | Reservoir | Surface |
|  |  |  |  | 5m |
|  |  |  |  | 10m |
|  |  |  |  | 15m |
|  |  | S8 | River | Surface |
|  | Yingzidu | S9 | Reservoir | Surface |
|  |  |  |  | 5m |
|  |  |  |  | 10m |
|  |  | S10 | River | Surface |
| 2018-April | Pingzhai | S1 | River | Surface |
|  |  | S2 | Reservoir | Surface |
|  |  |  |  | 5m |
|  |  |  |  | 15m |
|  |  | S3 | River | Surface |
|  | Puding | S4 | River | Surface |
|  |  | S5 | River | Surface |
|  |  | S6 | River | Surface |
|  |  | S7 | Reservoir | Surface |
|  |  |  |  | 5m |
|  |  |  |  | 10m |
|  |  |  |  | 15m |
|  |  | S8 | River | Surface |
|  | Yingzidu | S9 | Reservoir | Surface |
|  |  |  |  | 5m |
|  |  |  |  | 15m |
|  |  | S10 | River | Surface |
| 2020-September | Yuqiao | YQ | Reservoir | Surface |
|  | Chaobaihe | CBH | Reservoir | Surface |
|  |  | CBH1 | River | Surface |
|  | Bolonghu | BLH | Reservoir | Surface |
|  | Donglihu | DLH | Reservoir | Surface |
|  | Haihe | HH | Reservoir | Surface |
|  | Tuanbowa | TBW | Reservoir | Surface |
|  |  | TBW1 | River | Surface |
|  | Yongdinghe | YDH | Reservoir | Surface |

**Table S3**. Algal F_v_/F_m_ and cell volume from data collection.

| Species Name | Shape | Calculated or referenced | Cell volume (µm^3^) | ESD (µm) | F_v_/F_m_ | Reference |
| --- | --- | --- | --- | --- | --- | --- |
| **Cyanophyta** |  |  |  |  |  |  |
| *Anabaena* | sphere | d=6.0 | 113 | 7.3 | 0.35 | Berman-Frank et al. 2007 |
| *Anabaena cylindrica* | sphere | d=4.2 | 39 | 5.1 | 0.49 | This study |
| *Anabaena* sp*.* | sphere | d=6.0 | 113 | 7.3 | 0.38 | Lesser 2008 |
| *Anabaena* sp. PCC7120 | sphere | d=6.0 | 113 | 7.3 | 0.37 | Wu et al. 2006 |
| *Aphanizomenon flos-aquae* | cylinder | Olenina et al. 2006 | 65 | 6.0 | 0.21 | Shi et al. 2009 |
| *Aphanizomenon flos-aquae* | cylinder | d=3.1, h=8.3 | 65 | 6.0 | 0.34 | This study |
| *Aphanizomenon* sp*.* | cylinder | Olenina et al. 2006 | 65 | 6.0 | 0.23 | Karlberg and Wulff 2012 |
| *Aphanizomenon* sp. DC01 | cylinder | Olenina et al. 2006 | 65 | 6.0 | 0.32 | Hu et al. 2008 |
| *Cyanothece* | prolate spheroid | d=26, h=35 | 12501 | 34.9 | 0.50 | Berman-Frank et al. 2007 |
| *Microcoleus vaginatus Gom.* | cylinder | d=3, h=7.9 | 51 | 5.6 | 0.35 | Chen et al. 2009 |
| *Microcystis aeruginosa* | sphere | d=4.1 | 39 | 5.1 | 0.56 | This study |
| *Microcystis aeruginosa* | sphere | d=4.6 | 51 | 5.6 | 0.50 | Huang et al. 2015 |
| *Microcystis aeruginosa* DH-M1 | sphere | d=4.6 | 51 | 5.6 | 0.42 | Xiao et al. 2012 |
| *Microcystis aeruginosa Kützing* | sphere | d=4.6 | 51 | 5.6 | 0.58 | Deblois and Juneau 2012 |
| *Microcystis aeruginosa Kützing* | sphere | d=4.6 | 51 | 5.6 | 0.58 | Deblois and Juneau 2012 |
| *Microcystis aeruginosa Kützing* | sphere | d=4.6 | 51 | 5.6 | 0.65 | Deblois and Juneau 2012 |
| *Microcystis aeruginosa Kützing* | sphere | d=4.6 | 51 | 5.6 | 0.56 | Deblois and Juneau 2012 |
| *Microcystis aeruginosa Qutz.* | sphere | d=4.6 | 51 | 5.6 | 0.41 | Ou et al. 2005 |
| *Microcystis aeruginosa* TH-M2 | sphere | d=4.6 | 51 | 5.6 | 0.44 | Xiao et al. 2012 |
| *Microcystis flos-aquae* FACHB1174 | sphere | d=4.6 | 51 | 5.6 | 0.33 | Xiao et al. 2012 |
| *Microcystis* sp. FACHB1027 | sphere | d=4.6 | 51 | 5.6 | 0.44 | Xiao et al. 2012 |
| *Microcystis viridis* DC-M2 | sphere | d=4.6 | 51 | 5.6 | 0.36 | Xiao et al. 2012 |
| *Microcystis wesenbergii* DC-M1 | sphere | d=4.6 | 51 | 5.6 | 0.47 | Xiao et al. 2012 |
| *Microcystis-*FACHB 905 | sphere | d=4.6 | 51 | 5.6 | 0.30 | Shen and Song 2007 |
| *Microcystis-*FACHB 909 | sphere | d=4.6 | 51 | 5.6 | 0.47 | Shen and Song 2007 |
| *Microcystis-*FACHB 938 | sphere | d=4.6 | 51 | 5.6 | 0.48 | Shen and Song 2007 |
| *Microcystis-*FACHB 939 | sphere | d=4.6 | 51 | 5.6 | 0.53 | Shen and Song 2007 |
| *Microcystis*-FACHB 942 | sphere | d=4.6 | 51 | 5.6 | 0.35 | Shen and Song 2007 |
| *Microcystis-*FACHB 975 | sphere | d=4.6 | 51 | 5.6 | 0.51 | Shen and Song 2007 |
| *Microcystis-*PCC 7806 | sphere | d=4.6 | 51 | 5.6 | 0.36 | Shen and Song 2007 |
| *Microcystis-*PCC 7820 | sphere | d=4.6 | 51 | 5.6 | 0.40 | Shen and Song 2007 |
| *Nodularia spumigena* | cylinder | d=18, h=3 | 523 | 12.1 | 0.43 | Karlberg and Wulff 2012 |
| *Nostoc* sp. | cylinder | d=4.0, h=4.9 | 58 | 5.8 | 0.56 | This study |
| *Oscillatoria lutea var.contorta* | cylinder | d=4.4, h=4.4 | 65 | 6.0 | 0.44 | This study |
| *Pseudanabaena* sp*.* | cylinder | d=2.6, h=6.7 | 33 | 4.8 | 0.38 | This study |
| *Synechococcus elongatus* | sphere | d=7.6 | 230 | 9.2 | 0.26 | This study |
| **Cryptophyta** |  |  |  |  |  |  |
| *Cryptomonas erosa* | ellipsoid | a=32, b=31, h=24 | 9629 | 32.0 | 0.61 | Choi et al. 2012 |
| *Cryptomonas curvata* | prolate spheroid | d=32, h=24 | 9629 | 32.0 | 0.67 | This study |
| *Storeatula major* | ellipsoid | a=6, b=4, h=9 | 113 | 7.3 | 0.57 | Suggett et al. 2009 |
| *Storeatula major* | ellipsoid | a=6, b=4, h=9 | 113 | 7.3 | 0.57 | Suggett et al. 2009 |
| *Storeatula major* | ellipsoid | a=6, b=4, h=9 | 113 | 7.3 | 0.55 | Suggett et al. 2009 |
| **Bacillariophyta** |  |  |  |  |  |  |
| *Asterionella formosa* | box + 2 cylinders | Olenina et al. 2006 | 623 | 12.8 | 0.63 | Choi et al. 2012 |
| *Asterionella glacialis* | box + 2 cylinders | Olenina et al. 2006 | 623 | 12.8 | 0.479 | McMinn et al. 2005 |
| *Asterionella* sp*.* | box + 2 cylinders | Olenina et al. 2006 | 623 | 12.8 | 0.699 | McMinn et al. 2005 |
| *Biddulphia* sp. | elliptic prism | a=72, b=30, c=58 | 196965 | 87.5 | 0.598 | McMinn et al. 2005 |
| *Chaetoceros brevis* | elliptic prism | Olenina et al. 2006 | 195 | 8.7 | 0.46 | Timmermans et al. 2001 |
| *Chaetoceros brevis* | elliptic prism | Olenina et al. 2006 | 195 | 8.7 | 0.659 | Van de Poll et al. 2011 |
| *Chaetoceros brevis* | elliptic prism | Olenina et al. 2006 | 195 | 8.7 | 0.495 | Van de Poll et al. 2011 |
| *Chaetoceros calcitrans* | elliptic prism | a=15, b=4.0, c=2.0 | 195 | 8.7 | 0.62 | Şirin et al. 2015 |
| *Chaetoceros dichaeta* | elliptic prism | Olenina et al. 2006 | 195 | 8.7 | 0.41 | Timmermans et al. 2001 |
| *Chaetoceros mulleri* | elliptic prism | Olenina et al. 2006 | 195 | 8.7 | 0.65 | Antal et al. 2009 |
| *Chaetoceros socialis* | elliptic prism | Olenina et al. 2006 | 195 | 8.7 | 0.605 | McMinn et al. 2005 |
| *Coscinodiscus radiatus* | cylinder | López-Sandoval et al. 2014 | 82406 | 65.4 | 0.65 | López-Sandoval et al. 2014 |
| *Coscinodiscus* sp. | cylinder | d=17, h=42 | 9629 | 32.0 | 0.63 | Loebl et al. 2010 |
| *Coscinodiscus wailesii* | cylinder | López-Sandoval et al. 2014 | 2490327 | 203.8 | 0.71 | López-Sandoval et al. 2014 |
| *Cylindrotheca fusiformis* | prolate spheroid + 2 cylinders | d=6.0, h=7.0 | 589 | 12.6 | 0.79 | Lavaud et al. 2004 |
| *Detonula confervacea* | cylinder | d=8.0, h=20 | 1047 | 15.3 | 0.609 | McMinn et al. 2005 |
| *Ditylum brightwellii* | prism on triangle | l=45, m=30, h=64 | 43375 | 52.8 | 0.82 | Lavaud et al. 2004 |
| *Ditylum brightwellii* | prism on triangle | López-Sandoval et al. 2014 | 76162 | 63.7 | 0.60 | López-Sandoval et al. 2014 |
| *Fragilariopsis curta* | elliptic prism | Olenina et al. 2006 | 659 | 13.1 | 0.56 | Pankowski and McMinn 2009 |
| *Fragilariopsis cylindrus* | elliptic prism | Olenina et al. 2006 | 659 | 13.1 | 0.65 | Pankowski and McMinn 2009 |
| *Licmophora* spp. | gomphonemoid | Olenina et al. 2006 | 998 | 15.0 | 0.69 | McMinn et al. 2005 |
| *Melosira nummuloides* | cylinder | López-Sandoval et al. 2014 | 2308 | 19.9 | 0.62 | López-Sandoval et al. 2014 |
| *Melosira* sp. | cylinder | d=12, h=15 | 1697 | 17.9 | 0.632 | McMinn et al. 2005 |
| *Navicula pelliculosa* | elliptic prism | Olenina et al. 2006 | 555 | 12.4 | 0.63 | Kim et al. 2012 |
| *Navicula pelliculosa* | elliptic prism | Olenina et al. 2006 | 555 | 12.4 | 0.62 | Choi et al. 2012 |
| *Navicula* spp*.* | elliptic prism | Olenina et al. 2006 | 555 | 12.4 | 0.645 | McMinn et al. 2005 |
| *Navicula* spp*.* | elliptic prism | Olenina et al. 2006 | 555 | 12.4 | 0.546 | McMinn et al. 2005 |
| *Phaeodactylum* EPV | cylinder | d=8, h=20 | 950 | 14.8 | 0.42 | Kim et al. 2012 |
| *Phaeodactylum tricornutum* | cylinder | d=8, h=20 | 950 | 14.8 | 0.77 | Lavaud et al. 2004 |
| *Phaeodactylum tricornutum* | cylinder | López-Sandoval et al. 2014 | 92 | 6.8 | 0.63 | López-Sandoval et al. 2014 |
| *Pseudo-nitzschia* spp. | prism on parallelogram | a=90, b=8.0, h=2.0 | 735 | 13.6 | 0.608 | McMinn et al. 2005 |
| *Skeletonema costatum* | cylinder + 2 half spheres | d=20, h=28 | 13029 | 35.4 | 0.77 | Lavaud et al. 2004 |
| *Stephanodiscus* sp. | cylinder | Olenina et al. 2006 | 1098 | 15.5 | 0.66 | This study |
| *Thalassionema nitzschioides* | box | a=45, b=10, h=8.0 | 3590 | 23.0 | 0.621 | McMinn et al. 2005 |
| *Thalassiosira nordenskioeldii* | cylinder | d=24, h=36 | 15591 | 37.6 | 0.687 | McMinn et al. 2005 |
| *Thalassiosira nordenskioeldii* | cylinder | d=24, h=36 | 15591 | 37.6 | 0.75 | McMinn et al. 2005 |
| *Thalassiosira pseudonana* | cylinder | d=24, h=36 | 15591 | 37.6 | 0.62 | Loebl et al. 2010 |
| *Thalassiosira rotula* | cylinder | López-Sandoval et al. 2014 | 2571 | 20.6 | 0.62 | López-Sandoval et al. 2014 |
| *Thalassiosira* sp. | cylinder | d=24, h=36 | 15591 | 37.6 | 0.511 | McMinn et al. 2005 |
| *Thalassiosira weissflogii* | cylinder | d=24, h=36 | 15591 | 37.6 | 0.72 | Antal et al. 2009 |
| *Thalassiosira weissflogii* | cylinder | d=24, h=36 | 15591 | 37.6 | 0.79 | Lavaud et al. 2004 |
| *Thalassiosira weissflogii* | cylinder | d=24, h=36 | 15591 | 37.6 | 0.56 | Suggett et al. 2009 |
| *Thalassiosira weissflogii* | cylinder | d=24, h=36 | 15591 | 37.6 | 0.53 | Suggett et al. 2009 |
| *Thalassiosira weissflogii* | cylinder | López-Sandoval et al. 2014 | 1150 | 15.7 | 0.69 | López-Sandoval et al. 2014 |
| **Pyrrophyta** |  |  |  |  |  |  |
| *Akashiwo sanguinea* | sphere | López-Sandoval et al. 2014 | 47056 | 54.3 | 0.50 | López-Sandoval et al. 2014 |
| *Alexandrium minutum* | ellipsoid | López-Sandoval et al. 2014 | 5572 | 26.7 | 0.60 | López-Sandoval et al. 2014 |
| *Alexandrium tamarense* | ellipsoid | López-Sandoval et al. 2014 | 88983 | 67.1 | 0.57 | López-Sandoval et al. 2014 |
| *Amphidinium carterae* | ellipsoid | a=10, b=5.0, h=20 | 523 | 12.1 | 0.60 | Franklin and Berges 2004 |
| *Amphidinium carterae* | ellipsoid | a=10, b=5.0, h=20 | 523 | 12.1 | 0.60 | Antal et al. 2009 |
| *Amphidinium* sp. | ellipsoid | a=10, b=5.0, h=20 | 523 | 12.1 | 0.54 | Kim et al. 2012 |
| *Ceratium hirundinella* | ellipsoid + 2 cones + cylinder | Olenina et al. 2006 | 33493 | 48.5 | 0.59 | Whittington et al. 2000 |
| *Cochlodinium polykrikoides* | prolate spheroid | d=25, h=30 | 9850 | 32.2 | 0.43 | Kim et al. 2012 |
| *Prorocentrum minimum* | ellipsoid | a=10, b=18, h=20 | 1911 | 18.7 | 0.66 | Antal et al. 2009 |
| *Prorocentrum minimum* | ellipsoid | a=10, b=18, h=20 | 1911 | 18.7 | 0.54 | Suggett et al. 2009 |
| *Prorocentrum minimum* | ellipsoid | a=10, b=18, h=20 | 1911 | 18.7 | 0.51 | Suggett et al. 2009 |
| *Prorocentrum minimum* | ellipsoid | a=10, b=18, h=20 | 1911 | 18.7 | 0.46 | Suggett et al. 2009 |
| *Protoceratium reticulatum* | sphere | López-Sandoval et al. 2014 | 24012 | 43.4 | 0.61 | López-Sandoval et al. 2014 |
| *Peridinium umbonatum var. inaequale* | sphere | d=34.2 | 20934 | 41.4 | 0.45 | This study |
| **Chlorophyta** |  |  |  |  |  |  |
| *Ankistrodesmus* sp. | cylinder + 2 cones | d=25.9, h=4.8 | 950 | 14.8 | 0.70 | This study |
| *Chlamydomonas reinhardtii* | sphere | d=14 | 1436 | 17.0 | 0.73 | Antal et al. 2009 |
| *Chlamydomonas reinhardtii* | sphere | d=14 | 1436 | 17.0 | 0.63 | Choi et al. 2012 |
| *Chlamydomonas reinhardtii* | sphere | d=8.4 | 310 | 10.2 | 0.74 | This study |
| *Chlorella pyrenoidosa* | sphere | d=4.2 | 39 | 5.1 | 0.76 | Maksimova et al. 2004 |
| *Chlorella pyrenoidosa* | sphere | d=5.0 | 65 | 6.0 | 0.69 | This study |
| *Chlorella sorokiniana* | sphere | d=4.2 | 39 | 5.1 | 0.73 | Zhang et al. 2013 |
| *Chlorella vulgaris* | sphere | d=4.2 | 39 | 5.1 | 0.75 | Maxwell et al. 1994 |
| *Closterium* sp. | cylinder + 2 cones | d=322.0, h=25.0 | 142398 | 78.5 | 0.73 | This study |
| *Dunaliella tertiolecta* | prolate spheroid | d=12, h=9.0 | 589 | 12.6 | 0.60 | Vassiliev et al. 1995 |
| *Dunaliella tertiolecta* | prolate spheroid | d=12, h=9.0 | 589 | 12.6 | 0.72 | Berden-Zrimec et al. 2008 |
| *Dunaliella tertiolecta* | prolate spheroid | d=12, h=9.0 | 589 | 12.6 | 0.57 | Suggett et al. 2009 |
| *Dunaliella tertiolecta* | prolate spheroid | d=12, h=9.0 | 589 | 12.6 | 0.54 | Suggett et al. 2009 |
| *Dunaliella tertiolecta* | prolate spheroid | d=12, h=9.0 | 589 | 12.6 | 0.54 | Suggett et al. 2009 |
| *Haematococcus pluvialis* | sphere | d=26.4 | 9629 | 32.0 | 0.66 | This study |
| *Micromonas pusilla* | ellipsoid | López-Sandoval et al. 2014 | 11 | 3.3 | 0.59 | López-Sandoval et al. 2014 |
| *Nannochloropsis gaditana* | sphere | López-Sandoval et al. 2014 | 9 | 3.1 | 0.57 | López-Sandoval et al. 2014 |
| *Oocystis* sp. | prolate spheroid | d=17.8, h=9.6 | 860 | 14.3 | 0.68 | This study |
| *Ostreococcus tauri* | sphere | López-Sandoval et al. 2014 | 2 | 1.9 | 0.62 | López-Sandoval et al. 2014 |
| *Protococcus viridis* | sphere | d=5.7 | 102 | 7.0 | 0.73 | This study |
| *Pseudokirchneriella subcapitata* | ellipsoid | Olenina et al. 2006 | 51 | 5.6 | 0.69 | Choi et al. 2012 |
| *Pycnococcus provasolii* | sphere | d=2.0 | 4 | 2.4 | 0.41 | Suggett et al. 2009 |
| *Pycnococcus provasolii* | sphere | d=2.0 | 4 | 2.4 | 0.42 | Suggett et al. 2009 |
| *Pycnococcus provasolii* | sphere | d=2.0 | 4 | 2.4 | 0.33 | Suggett et al. 2009 |
| *Scenedesmus bijuga* | prolate spheroid | d=17.8, h=10.8 | 904 | 14.5 | 0.69 | This study |
| **Chrysophyta** |  |  |  |  |  |  |
| *Calcidiscus leptoporus* | sphere | López-Sandoval et al. 2014 | 51 | 5.6 | 0.67 | López-Sandoval et al. 2014 |
| *Emiliania huxleyi* | cylinder | d=3.0, h=0.3 | 2 | 1.9 | 0.57 | Loebl et al. 2010 |
| *Emiliania huxleyi* | cylinder | d=3.0, h=0.3 | 2 | 1.9 | 0.61 | Feng et al. 2008 |
| *Emiliania huxleyi* | cylinder | López-Sandoval et al. 2014 | 165 | 8.2 | 0.64 | López-Sandoval et al. 2014 |
| *Gephyrocapsa oceanica* | cylinder | López-Sandoval et al. 2014 | 82 | 6.5 | 0.66 | López-Sandoval et al. 2014 |
| *Isochrysis galbana* | sphere | López-Sandoval et al. 2014 | 58 | 5.8 | 0.68 | López-Sandoval et al. 2014 |
| *Isochrysis galbana* | sphere | d=5.4 | 82 | 6.5 | 0.57 | This study |
| *Pavlova lutheri* | ellipsoid | López-Sandoval et al. 2014 | 45 | 5.3 | 0.62 | López-Sandoval et al. 2014 |
| *Phaeocystis antarctica* | sphere | d=6.0 | 113 | 7.3 | 0.702 | Van de Poll et al. 2011 |
| *Phaeocystis antarctica* | sphere | d=6.0 | 113 | 7.3 | 0.573 | Van de Poll et al. 2011 |
| *Phaeocystis sp.* | sphere | d=6.0 | 113 | 7.3 | 0.628 | McMinn et al. 2005 |
| *Prymnesium parvum* | sphere | d=5.3 | 82 | 6.5 | 0.64 | This study |
| *Synura petersenii* | cone + half sphere | d=20, h=30 | 5422 | 26.4 | 0.62 | Choi et al. 2012 |
| **Phaeophyta** |  |  |  |  |  |  |
| *Aureococcus anophagefferens* | sphere | d=1.4 | 1 | 1.5 | 0.52 | Suggett et al. 2009 |
| *Aureococcus anophagefferens* | sphere | d=1.4 | 1 | 1.5 | 0.50 | Suggett et al. 2009 |
| *Nannochloropsis oculata* | sphere | d=4.0 | 33 | 4.8 | 0.58 | Kim et al. 2012 |
| *Chattonella marina* | cone + half sphere | d=22, h=48 | 8780 | 31.0 | 0.46 | Kim et al. 2012 |
| *Heterosigma akashiwo* | truncated cone & halfsphere | d=8.0, h=10 | 268 | 9.7 | 0.45 | Kim et al. 2012 |
| **Rhodophyta** |  |  |  |  |  |  |
| *Porphyridium purpareum* | sphere | d=5.2 | 74 | 6.3 | 0.40 | This study |
| *Rhodella reticulata* | sphere | d=5.6 | 92 | 6.8 | 0.55 | This study |
| **Euglenophyta** |  |  |  |  |  |  |
| *Euglena gracilis* | half ellipsoid + cone on ellitpic base | d=32.3, h=16.3 | 4443 | 24.7 | 0.54 | This study |

d, diameter; h, height; a, apical axis (length); b, transapical (width); l, length of one side; m, height of a triangle; ESD, Equivalent spherical diameter, calculated by cell volume. Calculated: cell volume was calculated by the parameters determined by light microscopy. Referenced: cell volume was cited from the literature. Biovolume calculation was referred to Hillebrand *et al.* (1999).

**Table S4**. Fragments per kilobase of transcription per million fragments mapped (FPKM) of genes of proteins involved in Photosystem Ⅱ, energy synthesis, carbon fixation, cytoskeleton, and cell wall of *Chlamydomonas reinhardtii* under different CO_2_ treatments.

|  |  | FPKM | | | | | |
| --- | --- | --- | --- | --- | --- | --- | --- |
|  |  | Initial CO_2_ concentration (μmol L^-1^) | | | | | |
| Pathway | Gene name | 3^a^ | 14^a^ | 52^b^ | 175^b^ | 497^b^ | 804^b^ |
| Photosystem II | Photosystem II reaction center W protein | 894 | 500 | 537 | 372 | 603 | 897 |
|  | hydroxymethylbilane synthase | 23 | 2 | 1 | 1 | 7 | 18 |
|  | magnesium-protoporphyrin O-methyltransferase | 27 | 10 | 14 | 10 | 42 | 70 |
|  | chlorophyll(ide) b reductase | 4 | 5 | 4 | 5 | 6 | 8 |
|  | geranylgeranyl diphosphate | 85 | 7 | 7 | 5 | 36 | 62 |
|  | 7-hydroxymethyl chlorophyll a reductase | 6 | 2 | 2 | 2 | 4 | 7 |
|  | magnesium chelatase subunit H | 40 | 7 | 5 | 4 | 10 | 23 |
| Energy synthesis | H+-transporting ATPase subunit a | 29 | 20 | 18 | 19 | 32 | 17 |
|  | NADPH-ferrihemoprotein reductase | 16 | 15 | 13 | 17 | 14 | 12 |
| Carbon fixation | acetyl-CoA carboxylase carboxyl transferase subunit beta | 14 | 7 | 5 | 7 | 17 | 28 |
|  | acetyl-CoA carboxylase carboxyl transferase subunit alpha | 8 | 6 | 4 | 6 | 11 | 23 |
|  | acetyl-CoA carboxylase, biotin carboxylase subunit | 12 | 6 | 10 | 8 | 26 | 40 |
|  | omega-6 fatty acid desaturase | 3 | 1 | 1 | 1 | 1 | 0 |
|  | stearoyl-CoA desaturase | 5 | 5 | 5 | 6 | 10 | 15 |
|  | starch synthase | 3 | 2 | 1 | 3 | 4 | 6 |
| Cell wall | 1,3-beta-glucan synthase | 0.11 | 0.05 | 0.01 | 0.03 | 0.05 | 0.01 |
|  | ADP-glucose pyrophosphorylase small subunit | 120 | 83 | 88 | 90 | 134 | 180 |
|  | ADP-glucose pyrophosphorylase large subunit | 138 | 73 | 70 | 67 | 95 | 138 |
|  | UDP-sugar pyrophosphorylase | 7 | 3 | 3 | 3 | 2 | 3 |
| Cytoskeleton | inositol-hexakisphosphate | 2 | 2 | 1 | 3 | 2 | 2 |
|  | actin related protein 2/3 complex | 6 | 3 | 2 | 2 | 2 | 2 |
|  | kinesin family member 5 | 9 | 1 | 1 | 4 | 2 | 1 |

^a^, CO_2_ limitation condition; ^b^, CO_2_ repletion condition.

**Table S5**. Fragments per kilobase of transcription per million fragments mapped (FPKM) of genes of proteins involved in Photosystem Ⅱ, energy synthesis, carbon fixation, cytoskeleton, and cell wall of *Chlamydomonas reinhardtii* under different nitrate treatments.

|  |  | FPKM | | | | |
| --- | --- | --- | --- | --- | --- | --- |
|  |  | Initial nitrate concentration (μmol L^-1^) | | | | |
| Pathway | Gene name | 25^a^ | 50^a^ | 250^b^ | 500^b^ | 2941^b^ |
| Photosystem II | Photosystem II reaction center W protein | 126 | 200 | 529 | 1082 | 776 |
|  | hydroxymethylbilane synthase | 4 | 13 | 5 | 7 | 14 |
|  | magnesium-protoporphyrin O-methyltransferase | 5 | 6 | 18 | 44 | 22 |
|  | hydroxymethylbilane synthase | 0 | 0 | 3 | 3 | 4 |
|  | geranylgeranyl diphosphate | 4 | 17 | 14 | 47 | 24 |
|  | 7-hydroxymethyl chlorophyll a reductase | 1 | 2 | 5 | 5 | 5 |
|  | magnesium chelatase subunit H | 3 | 6 | 13 | 16 | 31 |
| Energy synthesis | H+-transporting ATPase subunit a | 35 | 49 | 25 | 20 | 19 |
|  | NADPH-ferrihemoprotein reductase | 17 | 19 | 14 | 13 | 16 |
| Carbon fixation | acetyl-CoA carboxylase carboxyl transferase subunit beta | 2 | 6 | 19 | 15 | 18 |
|  | acetyl-CoA carboxylase carboxyl transferase subunit alpha | 3 | 4 | 24 | 12 | 28 |
|  | acetyl-CoA carboxylase, biotin carboxylase subunit | 2 | 7 | 14 | 24 | 13 |
|  | omega-6 fatty acid desaturase | 3 | 3 | 1 | 1 | 1 |
|  | stearoyl-CoA desaturase | 2 | 3 | 14 | 7 | 15 |
|  | starch synthase | 1 | 2 | 3 | 4 | 2 |
| Cell wall | 1,3-beta-glucan synthase | 0.04 | 0.04 | 0.04 | 0.05 | 0.10 |
|  | ADP-glucose pyrophosphorylase small subunit | 13 | 36 | 127 | 117 | 114 |
|  | ADP-glucose pyrophosphorylase large subunit | 14 | 33 | 88 | 87 | 86 |
|  | UDP-sugar pyrophosphorylase | 5 | 5 | 3 | 3 | 6 |
| Cytoskeleton | inositol-hexakisphosphate | 1 | 1 | 4 | 2 | 4 |
|  | actin related protein 2/3 complex | 3 | 3 | 3 | 2 | 5 |
|  | kinesin family member 5 | 1 | 2 | 3 | 4 | 1 |

^a^, nitrate limitation condition; ^b^, nitrate repletion condition.

**Table S6.** The average gene expression levels (AGELs) of proteins involved in Photosystem Ⅱ, energy synthesis, carbon fixation, cytoskeleton, and cell wall of phytoplankton assemblage in Tianjin’s reservoirs. The site names are referred to Table S2.

|  |  | AGEL | | | | |
| --- | --- | --- | --- | --- | --- | --- |
| Site | *F_v_/F_m_* | Photosystem Ⅱ | Energy synthesis | Carbon fixation | Cell wall | Cytoskeleton |
| BLH | 0.70 | 495 | 51 | 450 | 77 | 44 |
| CBH1 | 0.52 | 445 | 118 | 1719 | 18 | 67 |
| CBH | 0.86 | 326 | 35 | 133 | 25 | 27 |
| HH | 0.62 | 458 | 71 | 277 | 92 | 36 |
| TBW1 | 0.81 | 609 | 203 | 1061 | 30 | 134 |
| TBW | 0.50 | 432 | 55 | 795 | 95 | 89 |
| YDH | 0.81 | 967 | 656 | 5274 | 15 | 21 |
| YQ | 0.55 | 315 | 61 | 269 | 94 | 36 |
| DLH | 0.58 | 628 | 107 | 1320 | 41 | 54 |

**Figure**


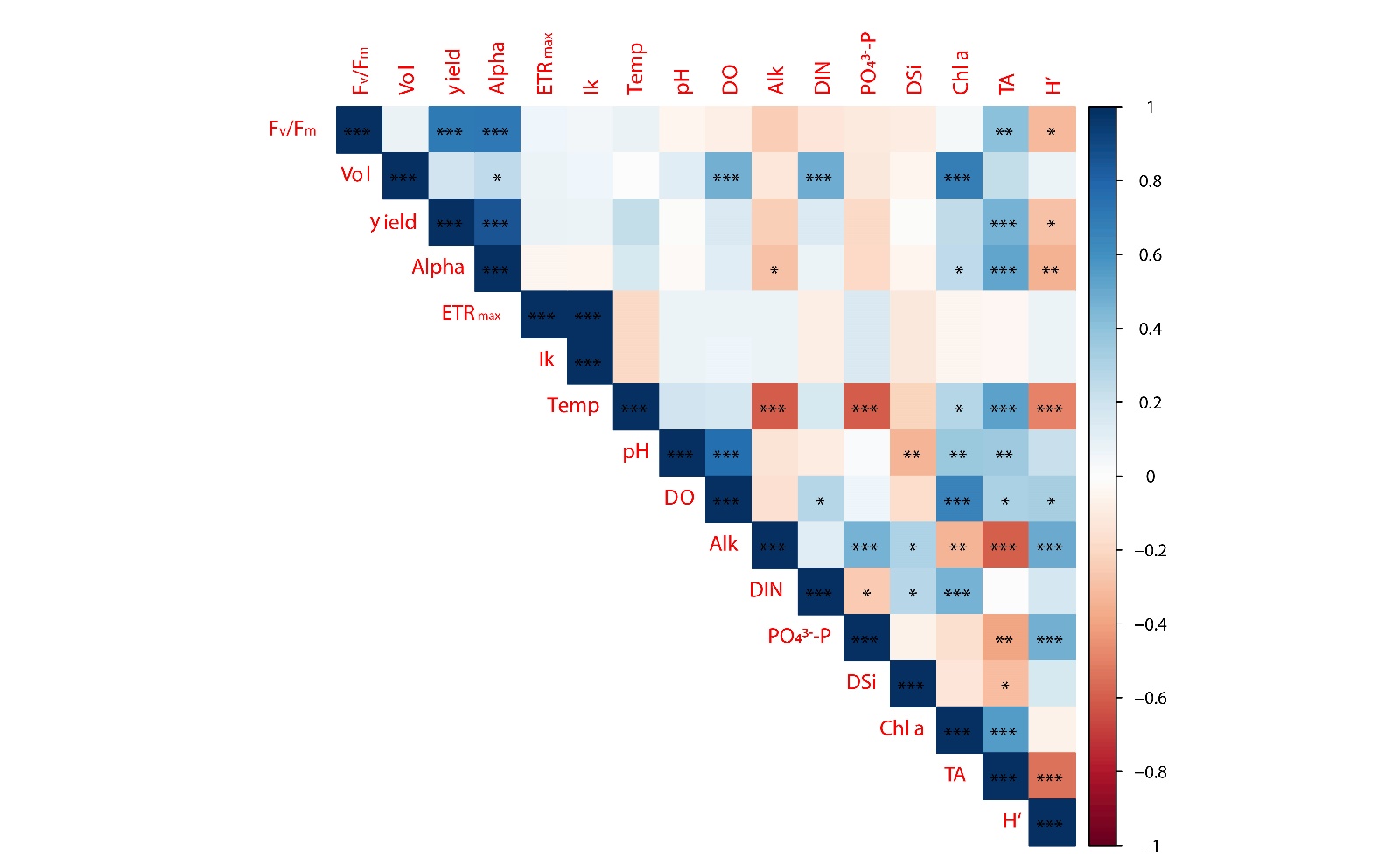


**Figure S1.** Results of Pearson correlation analysis in the field work. ***, ** and * are significant at the 0.001, 0.01 and 0.05 level, respectively. The color saturation denotes the correlation coefficient. F_v_/F_m_: maximum quantum yield, yield: effective quantum yield, Alpha: light use efficiency, ETR_max_: maximum electron transfer rate, I_k_: half-saturation light intensity, Temp: temperature, DO: dissolved oxygen, Alk: alkalinity, DIN: dissolved inorganic nitrogen, PO_4_^3-^-P: phosphate phosphorous, DSi: dissolved silicon, Chl *a*: chlorophyll *a* concentration, TA: total abundance, H՛: Shannon-Wiener index, Vol: mean cell volume of phytoplankton assemblage.


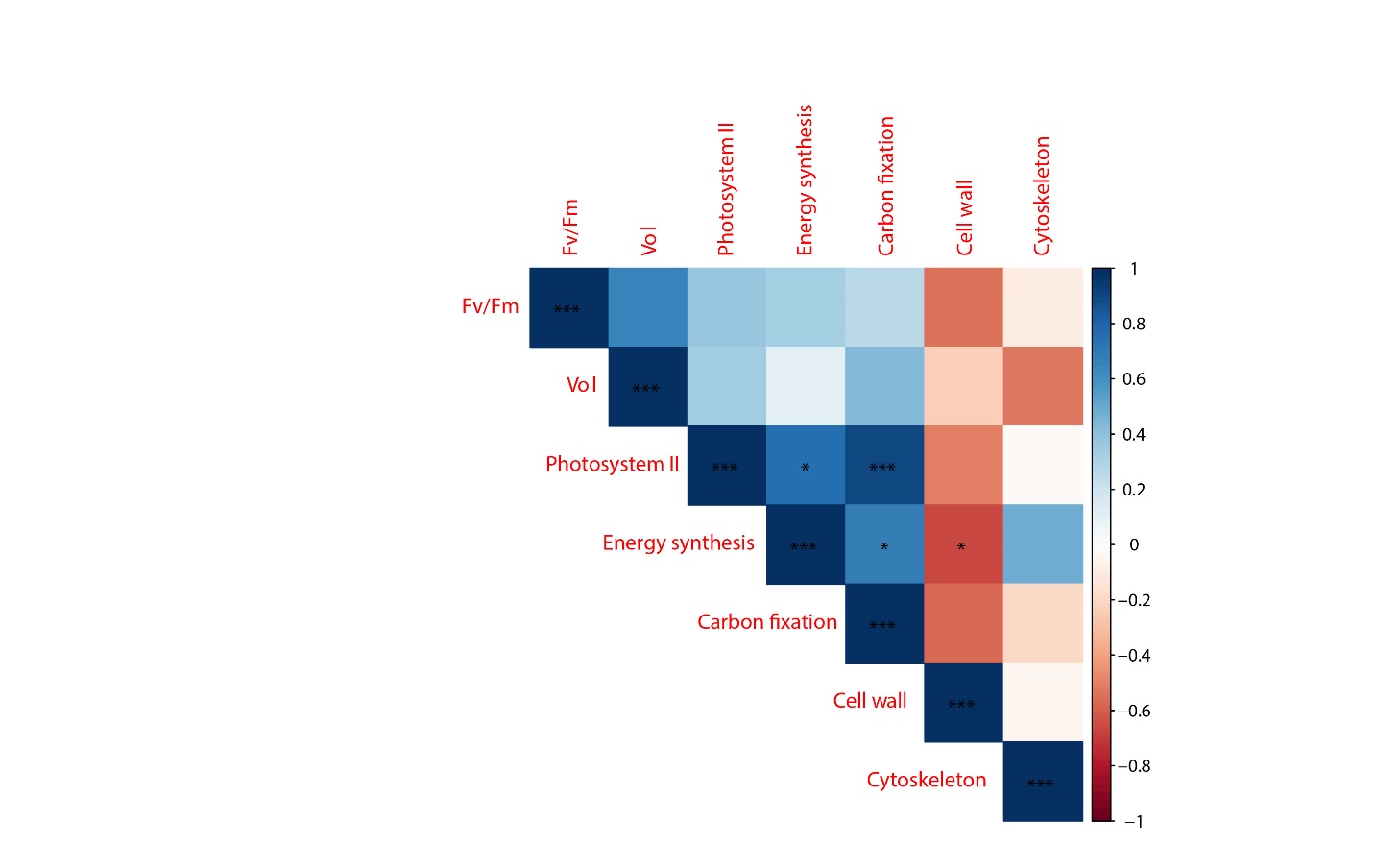


**Figure S2.** Results of Pearson correlation analysis for maximum quantum yield (F_v_/F_m_), mean cell volume of phytoplankton assemblage (Vol), and the average gene expression levels (AGELs) of proteins involved in Photosystem Ⅱ, energy synthesis, carbon fixation, cell wall, and cytoskeleton of samples in Tianjin’s reservoirs. ***, ** and * are significant at the 0.001, 0.01 and 0.05 level, respectively. The color saturation denotes the correlation coefficient.

**Figure S3.** Chlorophyll a (Chl *a*) density versus carbon density for phytoplankton assemblage in the field work. The regression: *y*=1.14*x*-1.09, adjusted *r^2^*=0.62, *p*<0.001.


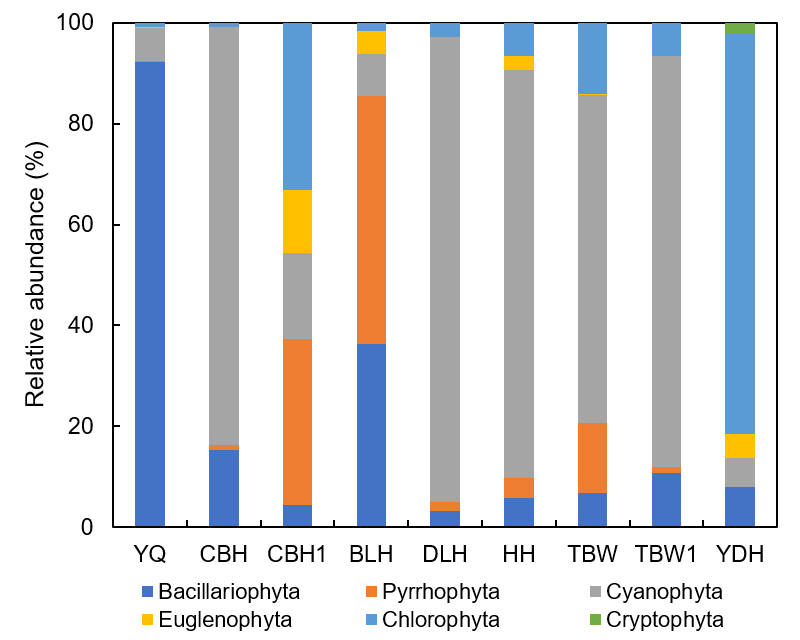


**Figure S4.** Phytoplankton community structure in Tianjin’s reservoirs. Relative abundance was calculated by the number of different algal phyla.

**Reference**

Antal, T. K., Matorin, D. N., Ilyash, L. V., Volgusheva, A. A., Osipov, V., Konyuhov, I. V., Krendeleva, T. E., and Rubin, A. B. 2009. Probing of photosynthetic reactions in four phytoplanktonic algae with a PEA fluorometer. *Photosynth. Res.* **102**: 67-76.

Berden-Zrimec, M., Drinovec, L., Molinari, I., Zrimec, A., Umani, S. F., and Monti, M. 2008. Delayed fluorescence as a measure of nutrient limitation in *Dunaliella tertiolecta*. *J. Photochem. Photobiol., B.* **92**: 13-18.

Berman-Frank, I., Quigg, A., Finkel, Z. V., Irwin, A. J., and Haramaty, L. 2007. Nitrogen-fixation strategies and Fe requirements in cyanobacteria. *Limnol. Oceanogr.* **52**: 2260-2269.

Chen, L. Z., Wang, G. H., Hong, S., Liu, A., Li, C., and Liu, Y. D. 2009. UV-B-induced oxidative damage and protective role of exopolysaccharides in desert cyanobacterium *Microcoleus vaginatus*. *J. Integr. Plant Biol.* **51**: 194-200.

Choi, C. J., Berges, J. A., and Young, E. B. 2012. Rapid effects of diverse toxic water pollutants on chlorophyll *a* fluorescence: variable responses among freshwater microalgae. *Water Res.* **46**: 2615-2626.

Deblois, C. P., and Juneau, P. 2012. Comparison of resistance to light stress in toxic and non‐toxic strains of *Microcystis aeruginosa* (cyanophyta). *J. Phycol*. **48**: 1002-1011.

Feng, Y., Warner, M. E., Zhang, Y., Sun, J., Fu, F. X., Rose, J. M., and Hutchins, D. A. 2008. Interactive effects of increased pCO_2_, temperature and irradiance on the marine coccolithophore *Emiliania huxleyi* (Prymnesiophyceae). *Eur. J. Phycol.* **43**: 87-98.

Franklin, D. J., and Berges, J. A. 2004. Mortality in cultures of the dinoflagellate *Amphidinium carterae* during culture senescence and darkness. *Proc. Biol. Sci.* **271**: 2099-2107.

Hillebrand, H., Claus-Diater [Dürselen](https://onlinelibrary.wiley.com/action/doSearch?ContribAuthorStored=D%C3%BCrselen%2C+Claus-Dieter), Kirschtel, D., Pollingher, U., and Zohary, T. 1999. Biovolume calculation for pelagic and benthic microalgae. *J. Phycol.* **35**: 403-424.

Hu, Z. Q., Li, D. H., Xiao, B., Dauta, A., and Liu, Y. D. 2008. Microcystin-RR induces physiological stress and cell death in the cyanobacterium *Aphanizomenon* sp. DC01 isolated from Lake Dianchi, China. *Fundam. Appl. Limnol.* **173**: 111-120.

Huang, H. M., Xiao, X., Ghadouani, A., Wu, J. P., Nie, Z. Y., Peng, C., Xu, X. H., and Shi, J. Y. 2015. Effects of natural flavonoids on photosynthetic activity and cell integrity in *Microcystis aeruginosa*. *Toxins* **7**: 66-80.

Karlberg, M., and Wulff, A. 2012. Impact of temperature and species interaction on filamentous cyanobacteria may be more important than salinity and increased pCO_2_ levels. *Mar. Biol.* **160**: 2063-2072.

Kim, Y. M., Wu, Y., Duong, T. U., Jung, S. G., Kim, S. W., Cho, H., and Jin, E. 2012. Algicidal activity of thiazolidinedione derivatives against harmful algal blooming species. *Mar. Biotechnol*. **14**: 312-322.

Lavaud, J., Rousseau, B., and Etienne, A. L. 2004. General features of photoprotection by energy dissipation in planktonic diatoms (Bacillariophyceae). *J. Phycol.* **40**: 130-137.

Lesser, M. P. 2008. Effects of ultraviolet radiation on productivity and nitrogen fixation in the cyanobacterium, *Anabaena* sp. (Newton's strain). *Hydrobiologia* **598**: 1-9.

Loebl, M., Cockshutt, A. M., Campbell, D. A., and Finkel, Z. V. 2010. Physiological basis for high resistance to photoinhibition under nitrogen depletion in *Emiliania huxleyi*. *Limnol. Oceanogr.* **55**: 2150-2160.

López-Sandoval, D. C., Rodríguez-Ramos, T., Cermeño, P., Sobrino, C., and Marañón, E. 2014. Photosynthesis and respiration in marine phytoplankton: relationship with cell size, taxonomic affiliation, and growth phase. *J. Exp. Mar. Biol. Ecol.* **457**: 151-159.

Maksimova, I. V., Bratkovskaya, L. B., and Plekhanov, S. E. 2004. Extracellular carbohydrates and polysaccharides of the alga *Chlorella pyrenoidosa* chick S-39. *Biol. Bull.* **31**: 175-181.

Maxwell, D. P., Falk, S., Trick, C. G., and Huner, N. P. A. 1994. Growth at low temperature mimics high-light acclimation in *Chlorella vulgaris*. *Plant Physiol.* **105**: 535-543.

McMinn, A., Hirawake, T., Hamaoka, T., Hattori, H., and Fukuchi, M. 2005. Contribution of benthic microalgae to ice covered coastal ecosystems in northern Hokkaido, Japan. *J. Mar. Biol. Assoc. U. K.* **85**: 283-289.

Olenina, I., Hajdu, S., Edler, L., Andersson, A., and Niemkiewicz, E. 2006. Biovolumes and size-classes of phytoplankton in the Baltic sea. *Helcom Balt. sea Environ. proc* **106**: 1-144.

Ou, M. M., Wang, Y., and Cai, W. M. 2005. Physiological and biochemical changes in *Microcystis aeruginosa* Qutz. in phosphorus limitation. *J. Integr. Plant Biol.* **47**: 692-702.

Pankowski, A., and McMinn, A. 2009. Iron availability regulates growth, photosynthesis, and production of ferredoxin and flavodoxin in Antarctic sea ice diatoms. *Aquat. Biol.* **4**: 273-288.

Shen, H., and Song, L. R. 2007. Comparative studies on physiological responses to phosphorus in two phenotypes of bloom-forming *Microcystis*. *Hydrobiologia* **592**: 475-486.

Shi, S. Y., Tang, D. S., and Liu, Y. D. 2009. Effects of an algicidal bacterium *Pseudomonas mendocina* on the growth and antioxidant system of *Aphanizomenon flos-aquae*. *Curr. Microbiol.* **59**: 107-112.

[Şirin](https://www.ncbi.nlm.nih.gov/pubmed/?term=%C5%9Eirin%20S%5BAuthor%5D&cauthor=true&cauthor_uid=25655268), S., Clavero, E., and [Salvadó](https://www.ncbi.nlm.nih.gov/pubmed/?term=Salvad%C3%B3%20J%5BAuthor%5D&cauthor=true&cauthor_uid=25655268), J. 2015. Efficient harvesting of *Chaetoceros calcitrans* for biodiesel production. *Environ. Technol*. **36**: 1902-1912.

Suggett, D. J., MacIntyre, H. L., Kana, T. M., and Geider, R. J. 2009. Comparing electron transport with gas exchange: parameterising exchange rates between alternative photosynthetic currencies for eukaryotic phytoplankton. *Aquat. Microb. Ecol.* **56**: 147-162.

Timmermans, K. R., Davey, M. S., Wagt, B., Snoek, J., Geider, R. J., Veldhuis, M. J. W., Gerringa, L. J. A., and Baar, H. J. W. 2001. Co-limitation by iron and light of *Chaetoceros brevis*, *C. dichaeta* and *C. calcitrans* (Bacillariophyceae). *Mar. Ecol. Prog. Ser.* **217**: 287-297.

Van de Poll, W. H., Lagunas, M., de Vries, T., Visser, R. J. W., and Buma, A. G. J. 2011. Non-photochemical quenching of chlorophyll fluorescence and xanthophyll cycle responses after excess PAR and UVR in *Chaetoceros brevis*, *Phaeocystis antarctica* and coastal Antarctic phytoplankton. *Mar. Ecol. Prog. Ser.* **426**: 119-131.

Vassiliev, I. R., Kolber, Z., Wyman, K. D., Mauzerall, D., Shukla, V. K., and Falkowski, P. G. 1995. Effects of iron limitation on photosystem II composition and light utilization in *Dunaliella tertiolecta*. *Plant Physiol.* **109**: 963-972.

Whittington, J., Sherman, B., Green, D., and Oliver, R. L. 2000. Growth of *Ceratium* *hirundinella* in a subtropical Australian reservoir: the role of vertical migration. *J. Plankton Res*. **22**: 1025-1045.

Wu, F., Yang, Z. L., and Kuang, T. Y. 2006. Impaired photosynthesis in phosphatidylglycerol-deficient mutant of cyanobacterium *Anabaena* sp. PCC7120 with a disrupted gene encoding a putative phosphatidylglycerophosphatase. *Plant Physiol.* **141**: 1274-1283.

Xiao, Y., Gan, N. Q., Liu, J., Zheng, L. L., and Song, L. R. 2012. Heterogeneity of buoyancy in response to light between two buoyant types of cyanobacterium *Microcystis*. *Hydrobiologia* **679**: 297-311.

Zhang, Y. M., Chen, H., He, C. L., and Wang, Q. 2013. Nitrogen starvation induced oxidative stress in an oil-producing green alga *Chlorella sorokiniana* C3. *PLoS One* **8**: 12.
